# Deciphering melatonin biosynthesis pathway in *Chenopodium quinoa*: genome-wide analysis and expression levels of the genes under salt and drought

**DOI:** 10.1007/s00425-025-04741-x

**Published:** 2025-06-12

**Authors:** Seher Yolcu, Ece Fidan, Muhammed Fatih Kaya, Emre Aksoy, Ismail Turkan

**Affiliations:** 1 Department of Soil Science and Plant Nutrition, Faculty of Agricultural Sciences and Technologies, Yasar University, Bornova, 35100 Izmir, Türkiye; 2 Faculty of Arts and Sciences, Department of Biological Sciences, Middle East Technical University, Ankara, Türkiye

**Keywords:** *Chenopodium quinoa*, Drought, Genome-wide analysis, Melatonin, Salt

## Abstract

**Main conclusion:**

In this study, we identified a total of ten melatonin biosynthesis genes (3 *TDCs*, 2 *TSHs*, 3 *SNATs*, and 2 *ASMTs*) in *Chenopodium quinoa* through bioinformatics methods, and analyzed physiological traits and gene expression levels in drought- and salt-treated plants with or without melatonin. Gene expression levels showed variations depending on tissues, genotypes, and abiotic stress.

**Abstract:**

Melatonin is involved in distinct biological processes, such as growth, development, and stress response in plants. The tryptophan decarboxylase (TDC), tryptamine 5-hydroxylase (T5H), serotonin N-acetyltransferase (SNAT), and N-acetylserotonin O-methyltransferase (ASMT) enzymes are involved in melatonin biosynthesis. Exogenous melatonin reduces the adverse effects of salt stress in different plants, but the roles of melatonin biosynthesis pathway in quinoa (*Chenopodium quinoa)* remain elusive. This study aims to identify and characterize the melatonin biosynthetic genes encoding TDCs, T5Hs, SNATs, and ASMTs in *C. quinoa* genome through bioinformatics methods and determine their transcript abundances under salt and drought stress. In total, ten genes were identified in *C. quinoa* genome, including 3 *TDCs*, 2 *TSHs*, 3 *SNATs*, and 2 *ASMTs*. TDCs have a pyridoxal-dependent decarboxylase domain, T5Hs possess a cytochrome P450, SNAT proteins contain the Acetyltransf_1 domain, and ASMTs include the O-methyltransferase domain. We also examined some physiological characteristics such as growth and water relations along with electrolyte leakage. For that purpose, two quinoa genotypes (Salcedo and Ames 1377) were subjected to salt and drought stress, with or without melatonin. Exogenous melatonin remarkably reduced the negative effects of salt and drought on shoot length, RWC, and electrolyte leakage in the sensitive Salcedo genotype. However, it showed limited impact on the stress-tolerant Ames 1377 genotype. Expression patterns showed variations depending on tissues, genotypes, and the type of abiotic stress. Promoter analysis indicated that the *cis-*elements in *TDC, T5H,* and *SNAT* promoters were mostly associated with stress-response, while those in *ASMT* promoters were related to light response.

**Supplementary Information:**

The online version contains supplementary material available at 10.1007/s00425-025-04741-x.

## Introduction

Melatonin (*N-*acetyl-5-methoxytryptamine), an endogenous compound, is present in various organisms, such as bacteria, protozoa, plants, fungi, and mammals (Dubbels et al. 1995; Tilden et al. 1997; Rodriguez-Naranjo et al. 2012; Ramasamy et al. 2023). Melatonin was discovered in different plants in 1995 through the use of radioimmunoassay and high-performance liquid chromatography (Hattori et al. 1995; Dubbels et al. 1995). It is a multifaceted compound extensively distributed across different plant organs, including leaves, roots, stems, flowers, fruits, and seeds (Ramasamy et al. 2023; Okazaki and Ezura 2009; Manchester et al. 2000). Melatonin is mainly produced in chloroplasts and mitochondria in different plant organs, including the leveas and roots, and can subsequently be transported to meristems, flowers, and fruits (Ayyaz et al. 2022). In tomato (*Solanum lycopersicum)*, melatonin was detected in various tissues including roots, leaves, stems, flowers, seeds, and fruits with concentrations ranging from 1.5 to 66.6 ng g^−1^ fresh weight (FW) (Okazaki and Ezura 2009). Melatonin functions as a growth regulator, bio-stimulator, and antioxidant (Kolodziejczyk and Kazmierczak 2024). It is involved in numerous physiological processes, such as seedling growth, the development of primary, lateral, and adventitious roots, flowering, enhancement of photosynthesis in response to leaf senescence, and seed development (Debnath et al. 2019). As an antioxidant, melatonin is known to decrease oxidative stress by scavenging reactive oxygen species (ROS), thereby protecting plant cells against environmental stress, such as drought, salt, UV-B radiation, and high temperature (Li et al. 2022; Khan 2023; Zhang et al. 2015; Afreen et al. 2006). When applied exogenously, melatonin has been shown to be absorbed through epidermal cells and transported via root-to-shoot translocation through the vascular system, playing a vital role in plant signaling and stress adaptation (Erland et al. 2019). Exogenous melatonin applications regulate germination, and elongation of roots in response to abiotic stress, such as osmotic and cold (Zhou et al. 2020; Wang et al. 2025). Previous studies have indicated that exogenous melatonin applications in plants can mitigate the negative impacts of abiotic stress factors, such as salinity and drought (Zhang et al. 2021; He et al. 2022; Liu et al. 2022; Song et al. 2024). For example, recently, melatonin-treated sugar beet *(Beta vulgaris)* seedlings improved growth performance under drought stress, by decreasing membrane lipid peroxidation, increasing activity of antioxidant enzymes, relative water content (RWC) in leaves, and chlorophyll synthesis (He et al. 2022). Liu et al. (2022) reported that the sugar beet seedlings under 60 µM of melatonin markedly enhanced the levels of soluble sugar and betaine. Exogenous melatonin applied to seeds could serve as an effective bio-stimulator, enhancing seed germination, plant growth, and crop production, especially under stressful conditions (Janas and Posmyk 2013). Under NaCl stress and melatonin treatment, a medicinal plant species, Japanese honeysuckle (*Lonicera japonica)* exhibited enhanced chlorophyll content, antioxidant enzyme activities, soluble sugar, and flavonoid concentrations, indicating improved tolerance to salt stress (Song et al. 2024). In tomato and rice (*Oryza sativa*) seedlings, melatonin treatment regulated the antioxidant system and increased proline levels under salt stress, leading to improved salt and drought tolerance and metabolic balance (Luo et al. 2022; Siddiqui et al. 2019). Interestingly, exogenous melatonin suppressed melatonin biosynthesis pathway while enhancing ROS-scavenging activities (Samadi et al. 2024; Pakgohar et al. 2024; Jahantighi et al. 2023). Furthermore, melatonin is considered highly useful in agriculture due to its multifaceted roles in plant physiology and stress tolerance (Janas and Posmyk 2013).

Melatonin biosynthesis pathway includes four major enzymes: tryptophan decarboxylase (TDC), tryptamine 5-hydroxylase (T5H), serotonin N-acetyltransferase (SNAT), and N-acetylserotonin O-methyltransferase (ASMT). The first enzyme, TDC, converts tryptophan to tryptamine, which is then synthesized into serotonin by T5H (Kang et al. 2007b, 2007a; De Masi et al. 2017; Wang et al. 2025). Serotonin is then converted to N-acetylserotonin by a third enzyme, SNAT (Kang et al. 2013), which is subsequently converted into N-acetyl-5-methoxytryptamine (melatonin) by the last enzyme, ASMT (Park et al. 2013). The crystal structure of rice TDC and its complexes with distinct ligands have been previously reported (Zhou et al. 2020). Zhang et al. (2024) recently discovered two *TDC* genes, *CsTDC1* and *CsTDC2*, in the cucumber (*Cucumis sativus*) genome, indicating their potential roles in growth, development, and stress response in cucumber. Transcript levels of *CsTDC1* and *CsTDC2* showed a significant increase in the seeds, but the lowest expression was observed in the old leaves of cucumber (Zhang et al. 2024). Various *TDC* genes in plants have been identified and their overexpression resulted in tolerance to multiple stresses, such as salt, drought, heavy metal, and pesticides (Zhao et al. 2019; Li et al. 2016; Wang et al. 2025). Under pesticide (fluroxypyr-meptyl) stress and melatonin application, TDC enzyme activity, the expression of differentially expressed genes (DEGs) and TDC-encoding genes increased in rice shoots and roots (Wang et al. 2025). Overexpression of *TDC* gene from herbaceous peony (*Paeonia lactiflora* Pall.) in tobacco plants resulted in a reduction of hydrogen peroxide (H_2_O_2_) and superoxide anion (O_2_^.-^), higher melatonin levels compared to the wild type, and consequently, increased tolerance to high salt and drought conditions (Zhao et al. 2019). So far, the *T5H* gene has been identified only in rice (Kang et al. 2007b). Through computational methods, *T5H* gene has also been identified in the tomato genome, sharing 55.3% identity with *OsT5H*. It is predicted to be localized in endoplasmic reticulum (Commisso et al. 2022). T5H is a soluble enzyme, which was found to have highest activity in the roots (Kang et al. 2007b). Tomato *TDC3 (SlTDC3)* was shown to work together with *SlT5H* to catalyze the conversion of tryptamine into serotonin in the fruits and roots (Commisso et al. 2022). Transgenic rice plants expressing human *SNAT* gene showed high SNAT enzyme activity, and an increase in the levels of serotonin and melatonin, chlorophyll synthesis under cold stress (Kang et al. 2010). Similarly, melatonin-rich transgenic rice expressing sheep *SNAT* gene exhibited higher biomass, delayed flower, and a reduction in grain yield under field conditions (Byeon and Back 2014). When *SNAT* gene expression was inhibited in rice, the synthesis of melatonin, seedling growth, and yield reduced, along with an increased sensitivity to abiotic stresses such as salt and cold (Byeon and Back 2016). The final enzyme in melatonin biosynthesis, a salt-tolerant apple (*Malus zumi) ASMT1 (MzASMT1),* was found to be upregulated by drought stress in leaves (Zuo et al. 2014). In tea (*Camellia sinensis)*, a total of 20 *ASMT* genes were identified and divided into three subfamilies according to phylogenetic analysis (Xu et al. 2023). Most *ASMT* genes did not exhibit alterations in response to cold and drought stresses, but the transcript levels of *CsASMT08, CsASMT09, CsASMT10*, and *CsASMT20* increased under low temperature and drought (Xu et al. 2023).

A facultative halophyte, quinoa (*Chenopodium quinoa*) which is a member of the Chenopodiaceae family, is a crucial crop species. Despite its tolerance for arid regions, drought or irrigation deficit negatively affects its growth (Pakgohar et al. 2024). There has been a significant increment in the production of quinoa, which was over 147 thousand metric tons in 2021 (Heitkam et al. 2020). Quinoa is an important source of high-quality proteins, lipids, amino acids, and mineral elements, such as Fe, Cu, Mg, and Zn. Furthermore, its seeds have a variety of saponins, anti-inflammatory compounds, and unsaturated fatty acids (Oshodi et al. 1999). Pakgohar et al. (2024) reported that foliar melatonin application in quinoa increased plant height, thousand grain weight, soluble carbohydrates, and reduced chlorophyll fluorescence under drought stress. In another study, the seed priming with melatonin in *C. quinoa* var. Titicaca led to an increase in dry weight, photosynthetic pigment levels, antioxidant enzyme activity, and a reduction in H_2_O_2_ content and lipid peroxidation under salt stress (Jahantighi et al. 2023). However, little is known about the relationship between melatonin biosynthesis pathway and abiotic stress response in quinoa. Plant genomes accomodate multiple genes for each of these four enzymes, all of which have not been identified yet and quinoa genome has no exception. Accordingly, to the best of our knowledge, melatonin biosynthetic genes, such as *TDC, T5H, SNAT,* and *ASMT,* have not been identified and characterized in quinoa yet through either computational or wet-lab methods. Therefore, in this study, four gene families (*TDC, T5H, SNAT,* and *ASMT*) involved in the melatonin biosynthesis pathway in quinoa have been identified and characterized based on their physical and chemical properties, phylogenetic relationships, subcellular localizations, conserved motifs, gene structures, protein–protein interaction, GO enrichment, and *cis-*acting regulatory elements in promoter regions. In addition, to validate the functions of melatonin biosynthesis, exogenous melatonin was applied to stress-sensitive (Salcedo) and tolerant (Ames 1377) quinoa genotypes under salt and drought stress. We examined the physiological parameters, and the transcript levels of the genes under these conditions. This study aims to provide an insight into the beneficial roles of melatonin under salt and drought stress, as well as the potential roles of TDCs, T5Hs, SNATs, and ASMTs in quinoa.

## Materials and methods

### Identification of genes encoding melatonin biosynthetic enzymes in quinoa

The TDC, T5H, SNAT, and ASMT amino acid sequences in rice and *Arabidopsis* were used to search quinoa proteins with the BLASTP tool (*C. quinoa* v1.0, Phytozome genome ID: 392, NCBI taxonomy ID: 63,459) in Phytozome (version 13; https://phytozome.jgi.doe.gov/pz/portal.html). All homologous protein sequences are accepted if they have sequence identity with *Arabidopsis* or rice TDC, T5H, SNAT, and ASMT proteins of more than 40% and e < 10^–10^. The TDC-, T5H-, SNAT-, and ASMT-specific domains, such as pyridoxal_deC, beta_elim_lyase, p450, acetyltransf_7, dimerisation, and methyltransf_2 in quinoa candidate proteins were confirmed by HMMER-based SMART (http://smart.embl-heidelberg.de/) (Letunic et al. 2012) and NCBI CDD (https://www.ncbi.nlm.nih.gov/cdd/) databases. Ten proteins’ coding sequences (CDS), promoter sequences, genomic, and amino acid sequences were retrieved from the Phytozome database. We have identified three TDCs, two T5Hs, three SNATs, and two ASMTs. The quinoa TDCs (TDC1-3), T5Hs (T5H1-2), SNATs (SNAT1-3), and ASMTs (ASMT1-2) were named based on their chromosomal localization. Physicochemical properties of 10 proteins involved in melatonin biosynthesis, such as isoelectric point (pI), theoretical molecular weight (MW), and GRAVY, were predicted online at the ExPASy server (https://web.expasy.org/protparam/) (Gasteiger et al. 2005).

### Subcellular localization prediction

Subcellular localizations of quinoa proteins involved in melatonin biosynthesis were predicted using two online predictors including the CELLO server (http://cello.life.nctu.edu.tw/) (Yu et al. 2006), and the WoLF PSORT Protein Subcellular Localization Prediction database (https://wolfpsort.hgc.jp/) (Nakai and Horton 1999; Horton et al. 2007).

### Phylogenetic analysis and genomic synteny

Phylogenetic trees of TDC, T5H, SNAT, and ASMT proteins in different plant species, including *A. thaliana (At), O. sativa (Os),* periwinkle* (Vinca minor) (Vm),* Madagascar periwinkle* (Catharanthus roseus, Cr),* devil pepper* (Rauvolfia verticillata, Rv),* toad tree* (Tabernaemontana elegans, Te),* kratom *(Mitragyna speciosa, Ms), Ophiorrhiza pumila (Op), Ophiorrhiza prostrata (Pr),* Chinese happy tree *(Camptotheca acuminata, Ca),* black cohosh *(Actaea racemosa, Ar),* barley* (Hordeum vulgare, Hv),* mulberry* (Morus notabilis, Mn),* sweet orange* (Citrus sinensis, Cs),* bean *(Phaseolus vulgaris, Pv),* castor bean* (Ricinus communis, Rc),* apple *(Malus domestica, Md),* cotton* (Gossypium arboreum, Ga),* wild soybean *(Glycine soja, Gs),* pigeonpea *(Cajanus cajan, Cc),* and salt-tolerant apple (*Malus zumi, Mz)* were constructed by MEGA11 (https://www.megasoftware.net/history.php) software with Neighbor-Joining (NJ) method (1000 bootstrap replicates) (Tamura et al. 2021).

Genomic synteny was comparatively performed to investigate the evolutionary relationship between quinoa and *Arabidopsis,* rice, perinwinkle*,* Madagascar periwinkle*,* Indian snake-root* (Rauvolfia verticillata, Rv),* crepe jasmine* (Tabernaemontana elegans, Te),* kratom*, O. pumila (Op), O. prostrata (Pr),* Chinese happy tree*,* black cohosh*,* barley*,* mulberry*,* sweet orange*,* bean*,* castor bean*,* apple*,* cotton*,* wild soybean*,* pigeonpea*,* and salt-tolerant apple amino acid sequences using the circoletto program (Circos) (tools.bat.inspire.org/circoletto/) (Krzywinski et al. 2009). Score/max ratio was used coloring with blue <  = 0.25, green <  = 0.50, orange <  = 0.75, red > 0.75. Ten protein sequences from quinoa and 63 from various plant species in FASTA format were included in the query and database file, respectively.

### Conserved motifs and structures of genes

The Multiple Expectation Maximization for Motif Elicitation (MEME) Suite tool (http://meme-suite.org/tools/meme) was used to determine conserved motifs of the quinoa TDC, T5H, SNAT, and ASMT proteins using with the following parameters: the maximum number of motifs is 10 and zero or one occurrence (of a contributing motif site) per sequence mode (Bailey et al. 2006). Gene Structure Display Server (GSDS) (http://gsds.gao-lab.org/) (Bo et al. 2015) was used to examine the exon–intron organizations of the TDC-, T5H-, SNAT-, and ASMT-encoding genes.

### *Cis*-acting regulatory elements in promoter regions

The sequences 1500 bp before the transcription start site were obtained from the quinoa genome using the Phytozome database. PlantCARE software predicted the numbers and types of *cis-*elements (http://bioinformatics.psb.ugent.be/webtools/plantcare/html/) (Lescot et al. 2002).

### Protein–protein interaction (PPI) network, clustering, and gene ontology enrichment analysis

*Arabidopsis* orthologs of *SNAT1, SNAT2, SNAT3, ASMT1, ASMT2*, and *T5H1* were identified using TAIR BLAST 2.9.0 + (Table S1). Orthologs of these genes were used to search the PPI networks through String version 12.0 (Szklarczyk et al. 2019) with the minimum required interaction score of 0.4. Then, the proteins in the network were clustered by k-means in two clusters (Likas et al. 2003). Finally, the genes in these clusters were used as a set to determine the gene ontology (GO) enrichment for biological process and molecular function using Fisher’s exact test with the Bonferroni correction for multiple testing (*p* < 0.01) (Consortium 2023) and visualized by String version 12.0 with a signal and a strength rate of ≥ 0.01 (Table S2).

### Growth conditions and stress applications

The seeds of two quinoa genotypes with contrasting tolerance levels, Ames 1377 (tolerant) and Salcedo (sensitive), to salinity and high temperature (Ain et al. 2023; Bunce 2017; Prajapat et al. 2024; Tovar et al. 2020; Dehghanian et al. 2024; Hinojosa et al. 2018) were used in the growth experiment conducted in a climate-controlled greenhouse. The Ames 1377 seeds, originating from New Mexico, USA, were obtained from the USDA, while the Salcedo seeds, originating from Puno, Peru, were provided by the Türkiye Quinoa Growers Association. The seeds were sown in 1 L plastic pots filled with a soil mixture (2:1:1 ratio of soil, peat, and perlite). At the start of the experiment, pots were hand-irrigated with distilled water until the soil reached 100% field capacity (θ_FC_), after which soil volumetric water content (θ_v_) was logged daily at 10 cm depth using capacitance probes (Sentek Sensor Technologies) and the pots were weighed and hand-watered every other day to restore θ_v_ to 0.20–0.22 m^3^ m⁻^3^ (70% θ_FC_), since literature indicates that quinoa achieves optimal water-use efficiency and biomass at 60–70% θ_FC_ (Sanandaji et al. 2024). After 2 weeks of growth at 22 ± 2 °C and 50% relative humidity in a climate-controlled greenhouse under long-day photoperiod, they were thinned to three uniform seedlings per pot. The experiment was designed according to a split-plot arrangement in a completely randomized design (CRD), where control and stress treatments were assigned to the main plots, while melatonin applications were assigned to the subplots. The pots represented biological replicates and replicated three times.

Stress treatments began 20 days after sowing. The plants were divided into five groups for each genotype: (i) control, (ii) salt stress (300 mM NaCl), (iii) salt stress with 70 µM melatonin, (iv) drought (40% field capacity, FC), and (v) drought with 70 µM melatonin. The control group was irrigated to maintain 100% FC throughout the experiment. In the salt-stressed groups, plants were irrigated with a 300 mM NaCl solution for 15 days. For salt stress combined with melatonin, 70 µM melatonin was added to the NaCl irrigation solution. For the drought-stressed group, irrigation was withheld until the soil FC dropped to 40%, after which it was maintained at 40% FC for 15 days. In the drought plus melatonin group, 70 µM melatonin was added to the irrigation water used to maintain 40% FC. All stress treatments lasted for 15 days. At the end of the stress applications, physiological measurements were done from the fully developed youngest leaves of the plants.

### Growth and physiological parameters

Shoot length and several physiological parameters, including the RWC and electrolyte leakage, were measured under drought and salt stress.

The shoot length of the plants was measured with a ruler from the shoot attachment point to the tip of the stem.

The harvested leaves were immediately weighed using a precision balance to record the fresh weights. Subsequently, fresh leaf samples were soaked in distilled water for 24 h at room temperature, and their turgid weights were measured and recorded. The turgid leaf samples were then dried in an oven at 65 °C for 48 h, after which their dry weights were measured and recorded. The relative water content (RWC) was calculated using the following formula:

$${\text{Relative}}\,{\text{Water}}\,{\text{Content}}\,(\% ) = \frac{{\left( {{\text{Fresh }}\,{\text{Weight}} - {\text{Dry }}\,{\text{Weight}}} \right) }}{{ \left( {{\text{Turgid }}\,{\text{Weight }} - {\text{Dry }}\,{\text{Weight}}} \right)}} \times 100.$$

The leaf samples were placed in falcon tubes containing 30 mL of deionized water. The samples were then incubated under long-day photoperiod conditions (16 h light/8 h dark) in a controlled climate chamber at 23 ± 1 °C, with 60% humidity and 500 lx light, for 24 h. After incubation, the electrical conductivity (EC) of the plant samples was measured using an EC meter. Subsequently, the plant samples were autoclaved at 121 °C for 10 min to break down the leaf tissues. Following autoclaving, the temperature of the mixture in the tubes was brought to 25 °C, and the EC values were measured again. Electrolyte leakage was calculated using the following formula (Bajji et al. 2002):

$$\% {\text{ Electrolyte Leakage}}\, = \,\left[ {\frac{{\left( {T2 {-} T1} \right)}}{{\left( {T3 - T1} \right)}}} \right]/FW \times 100,$$

where

T1: EC value before incubation under light.

T2: EC value after incubation under light.

T3: EC value after autoclaving.

FW: Fresh weight of the leaf.

### Total RNA isolation and quantitative real-time PCR (qRT-PCR)

The total RNA from quinoa roots and leaves were extracted using TriZOL according to Chomczynski and Sacchi (2006) with small modifications. RNA extraction was performed starting with the grinding of samples in liquid nitrogen to ensure effective cell disruption and preservation of RNA integrity. Immediately, 1 mL of TriZOL reagent was added to the ground samples, and the mixture was homogenized using vortexing. The samples were left to sit at room temperature for 15 min to increase the yield of cell lysis. Pre-cooled centrifuge conditions were prepared at 4 °C prior to further processing, and all the centrifugations were carried out on 20.000 x g. Ice-cold chloroform (250 µL) was then added to each sample, and the mixture was gently inverted for 15 s to ensure proper phase separation. After incubation at room temperature for 5 min, the samples were centrifuged for 30 min at 4 °C. Following centrifugation, 600 µL of the aqueous phase was carefully transferred to new Eppendorf tubes. To precipitate RNA, 200 µL of 3 M lithium chloride (LiCl) and 600 µL of ice-cold isopropanol were added and mixed by inverting the tubes up and down for 15 s. The samples were incubated overnight at − 20 °C to enhance RNA precipitation. The RNA was pelleted by centrifuging the samples at maximum speed for 30 min at 4 °C. The supernatant containing isopropanol was carefully removed without disturbing the RNA pellet. The pellet was then washed with ice-cold 75% ethanol, taking care to avoid direct contact with the pellet while ensuring resuspension. The samples were centrifuged again for 10 min at maximum speed at 4 °C, after which any residual ethanol was discarded with pipetting. The RNA pellet was air-dried by positioning the sample tubes perpendicularly on a rack for 2–3 min. Finally, the RNA pellet was resuspended with 50 µL DEPC-treated distilled water.

For first-strand cDNA synthesis, a reaction mixture was prepared using Transbiotech *EasyScript*® First-Strand cDNA Synthesis SuperMix Catalog Number:AE301-02. Random primer (0.1 µg/µL) was used to amplify the cDNA from total RNA samples. The reaction mixture included 1 µL of Random primer, 10 µL of 2 × ES Reaction Mix, 1 µL of EasyScript® RT/RI Enzyme Mix, and RNase-free water to bring the final volume to 20 µL. For improved synthesis efficiency with complex RNA templates, RNA template, primers, and RNase-free water were mixed and incubated at 65 °C for 5 min, followed by cooling on ice for 2 min before adding other components. The samples were initially incubated at 25 °C for 10 min, followed by 42 °C for 15 min for qPCR, as manufacturer suggests. Finally, the reaction was terminated by incubating samples at 85 °C for 5 s to inactivate the enzymes. In total, 100 ng of cDNA samples were synthesized for each sample. Gene-specific primers (Table S3) were used for targeted amplification in quantitative Real-Time PCR (qRT-PCR) to evaluate synthesized complementary DNA (cDNA) samples. In each reaction, 100 ng of cDNA was used, equivalent to 2 µL based on its concentration. The 25 µL reaction mixture included 12.5 µL of SYBR GREEN Master Mix (Ampliqon RealQPlus 2 × SYBR Green Master Mix, Cat No. 323402), 9.5 µL of nuclease-free PCR-grade water, 1 µL of gene-specific primers, and 2 µL of cDNA template. Two biological replicates were used and each biological replicate split into two technical replicates. The qRT-PCR protocol initiated with 15 min of denaturation at 95 °C to activate DNA polymerase and fully denaturate the DNA template. This was followed by 40 amplification cycles. Denaturation at 95 °C for 15 s separated the DNA strands, annealing for 20 s allowed the gene-specific primers bind to the target sequences, annealing step temperature was calculated for each primer set, and an extension step at 72 °C for 20 s synthesized corresponding DNA strands.

Quantitative RT-PCR data were analyzed using the ΔΔCt method as described by Muller et al. (2002). Relative expression levels were calculated by normalizing the Ct values of the target genes to those of the reference gene (housekeeping gene) in each sample (*ACTIN1* gene used as internal control). The resulting ΔCt values were then compared to a calibrator sample to obtain ΔΔCt values. Fold changes in gene expression were determined as 2^−ΔΔCt^, providing the relative expression of target genes in each sample.

### Statistical analysis

Data were analyzed using ANOVA in Minitab 17 (Minitab 2014). Mean differences were assessed by Tukey’s post hoc test at a 5% significance level within each genotype. Genotype × treatment interactions were analyzed via two-way ANOVA. Graphs were generated using GraphPad Prism 10.4.1 (GraphPad 2024).

## Results

### Identification of *TDC, T5H, SNAT, *and *ASMT* genes in quinoa, their subcellular localizations, and phylogenetic relationships

The protein sequences of TDC, T5H, SNAT, and ASMT from rice and *Arabidopsis *were retrieved from NCBI and Phytozome. These sequences were then used as queries to search for quinoa proteins using BLASTP. In total, ten genes in quinoa were found to encode 3 TDC, 2 T5H, 3 SNAT, and 2 ASMT proteins, which were named according to their chromosomal positions and enzymatic reactions as *CqTDC1-3, CqT5H1-2, CqSNAT1-3,* and *CqASMT1-2*. The ProtParam tool was employed to analyze the physicochemical features of TDCs, T5Hs, SNATs, and ASMTs. These properties included chromosomal location, strand, CDS (bp), amino acid lengths (aa), theoretical molecular weights (MW), isoelectric points (pI), and GRAVY, as shown in Table 1. For all genes and proteins detected in quinoa, the coding sequences (CDS) ranged from 315 base pairs (bp) in *CqSNAT3* to 1905 bp in *CqTDC2*, while the protein lengths varied from 105 to 635 amino acids (aa). The shortest amino acid lengths were found in SNAT proteins, while the longest were observed in TDC proteins. The highest molecular weight was observed in the CqTDC2 protein (71.96 kDa), while the lowest was seen in CqSNAT3 (11.55 kDa). The pI values varied significantly, ranging from 4.91 (SNAT1) to a maximum value of 9.69 (SNAT2). In contrast, the pI values of SNAT1 and SNAT3 were 4.91 and 5.47, respectively. Among the quinoa proteins involved in melatonin biosynthesis, seven—including TDC2, TDC3, T5H1, SNAT1, SNAT3, and ASMT1-2—are acidic, with SNAT1 being the most acidic one. All proteins, except for TDC1 and ASMT1, displayed negative GRAVY values, indicating their hydrophilic nature (Table 1).

**Table 1:** The physicochemical properties of *TDC, T5H, SNAT,* and *ASMT* genes in quinoa

Sequence ID Phytozome	Gene name	Domain(s)	Location	Strand	CDS (bp)	Length (aa)	MW (kDa)	pI	GRAVY
AUR62014902	*CqTDC1*	Pyridoxal phosphate-decarboxylase	Scaffold_2947:4,708,324–4713286	forward	1425	475	52.72	7.54	0.127
AUR62042693	*CqTDC2*	Pyridoxal phosphate-decarboxylase	Scaffold_3294:2,551,943–2,558,344	reverse	1905	635	71.96	5.47	− 0.130
AUR62025133	*CqTDC3*	Pyridoxal phosphate-decarboxylase	Scaffold_4082:6,076,253–6077792	reverse	1539	513	57.27	6.33	− 0.063
AUR62001217	*CqT5H1*	Cytochrome p450	Scaffold_2716:1,706,190–1708094	forward	1560	520	59.35	6.72	− 0.183
AUR62022106	*CqT5H2*	Cytochrome p450	Scaffold_4319:1,212,533–1,214,885	reverse	1581	527	60.25	7.60	− 0.211
AUR62006445	*CqSNAT1*	Acetyltransferase 7	Scaffold_1001:78,286,277,832,755	forward	597	199	21.76	4.91	− 0.266
AUR62007827	*CqSNAT2*	Acetyltransferase 1	Scaffold_2646:4,624,654–4,625,236	forward	582	194	21.64	9.69	− 0.143
AUR62029798	*CqSNAT3*	Acetyltransferase 7	Scaffold_2858:1,729,791–1,733,933	forward	315	105	11.55	5.47	− 0.239
AUR62012984	*CqASMT1*	Dimerisation/Methyltransferase 2	Scaffold_1040:963,159–964,741	forward	969	323	35.38	5.53	0.056
AUR62009996	*CqASMT2*	Dimerisation/Methyltransferase 2	Scaffold_1257:484,361–486,171	forward	1089	363	39.91	4.95	− 0.047

In this study, two online predictors, cello-life and WoLFPSORT, were utilized to predict the subcellular localization of quinoa proteins involved in melatonin biosynthesis (Table 2). Based on the findings from WoLFPSORT, the subcellular localizations of four proteins, including TDC1, SNAT3, ASMT1, and ASMT2, were predicted to be in the cytosol. Three proteins (SNAT3, ASMT1, and ASMT2) were consistently located within the cytoplasm based on cello-life. Additionally, SNAT2 was consistently found in the mitochondria according to both cello-life and WoLFPSORT predictions. Cello-life predicted plasma membrane localizations for TDC1-3 proteins, while WoLFPSORT putatively determined that TDC1 was cytosolic and TDC2-3 were found in the cytoskeleton. The WoLFPSORT tool predicted the chloroplastic localization of T5H1, T5H2, and SNAT1 proteins, while these proteins were assumed to be localized in the cytoplasm based on cello-life results. No proteins existed in the nucleus. Cello-life predicted 4 cytosolic and 3 chloroplastic proteins, while WoLFPSORT tool showed 3 proteins in plasma membrane, and 5 cytosolic and 1 mitochondrial protein. Different subcellular locations of SNATs may be associated with functional variation. In the current study, two online predictors demonstrated variations in subcellular locations of proteins involved in the melatonin biosynthesis pathway. Differences in subcellular localizations obtained from bioinformatics tools prompted us to conduct confirmatory studies using experimental methods.

**Table 2:** Predicted subcellular localization of TDC, T5H, SNAT, and ASMT proteins in quinoa*.* Two online prediction tools including cello-life, and WoLFPSORT were used to determine the subcellular localizations (*cyto: cytosol, cysk: cytoskeleton, nucl: nucleus, mito: mitochondrium, chlo: chloroplast*)

Quinoa Protein	Subcellular localization	
	Cello-life	WoLF PSORT
CqTDC1	Plasma membrane	cyto: 10, chlo: 2
CqTDC2	Plasma membrane	cysk: 6, nucl: 3
CqTDC3	Plasma membrane	cysk: 8, cyto: 3
CqT5H1	Cytoplasm/mitochondrium	chlo: 12, cyto: 1
CqT5H2	Cytoplasm/mitochondrium	chlo: 13, cyto: 1
CqSNAT1	Cytoplasm	chlo: 10, mito: 2
CqSNAT2	Mitochondrium	mito: 7.5, chlo_mito: 6.5
CqSNAT3	Cytoplasm	cyto: 8, nucl: 2
CqASMT1	Chloroplast/cytoplasm	cyto: 7, cysk: 6
CqASMT2	Cytoplasm	cyto: 7, cysk: 4

**Fig. 1:**
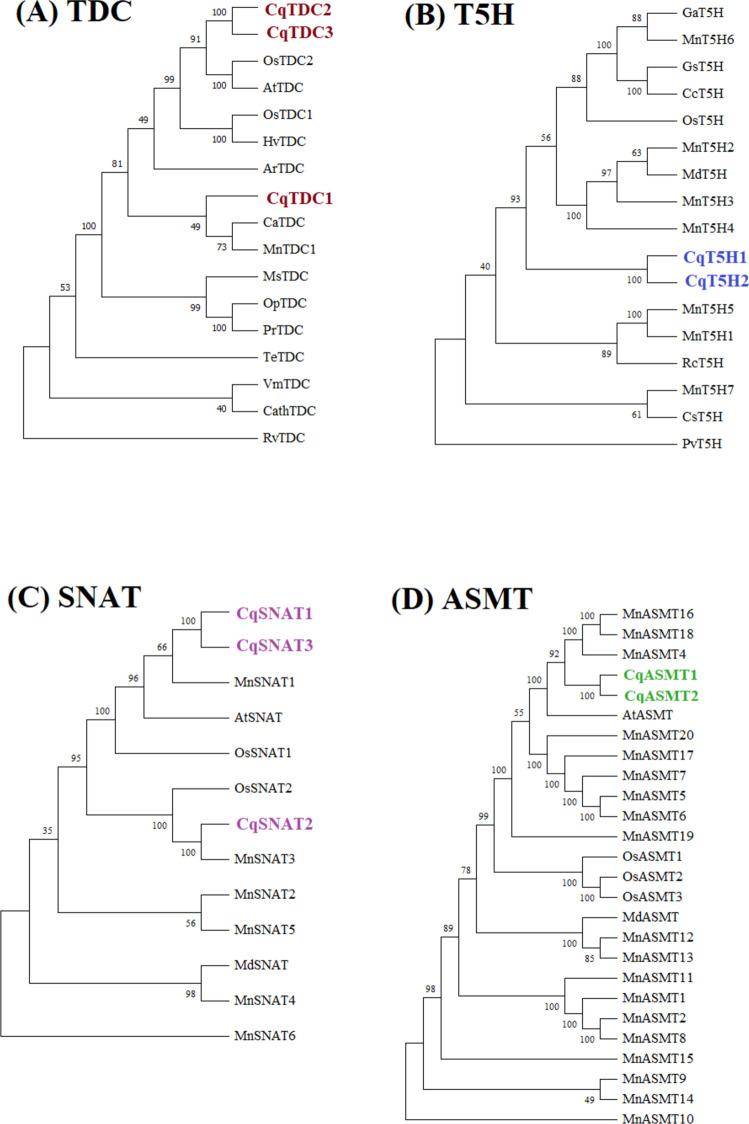
Phylogenetic trees of TDC (**A**), T5H (**B**), SNAT (**C**), and ASMT (**D**) amino acid sequences involved in melatonin biosynthesis in *C. quinoa* (Cq) and other plant species. These phylogenetic trees were generated using the neighbor-joining method with MEGA 11 software

Using the NJ method with 1000 bootstrap values, phylogenetic trees were generated from a total of 17 TDCs, 17 T5Hs, 13 SNATs, and 26 ASMT proteins from various plant species (Fig. 1). Quinoa proteins were represented by different colors. Based on the phylogenetic analysis, CqTDC2 and CqTDC3 appear closely related to OsTDC2 and AtTDC, while the CqTDC1 was located at the same clade with Chinese happy tree TDC (CaTDC) and mulberry TDC1 (MnTDC1) (Fig. 1A). A large clade comprising T5H proteins from different plant species including rice*,* mulberry*,* wild soybean*,* cotton*,* apple, and pigeonpea showed a close relationship with quinoa T5H1 and T5H2 sequences (Fig. 1B). The SNAT1 and SNAT3 were found at the same clade with mulberry SNAT1 (MnSNAT1), *Arabidopsis* SNAT (AtSNAT) and rice SNAT1 (OsSNAT1), while CqSNAT2 clustered with MnSNAT3 and OsSNAT2 (Fig. 1C). According to the findings of Zheng et al. (2021), 25 ASMT proteins from four plant species were categorized into three distinct classes (I–III) as shown in our phylogenetic analysis. Here, Class II proteins included CqASMT1-2, MnASMT4-16–18, and AtASMT. However, Class I and III did not contain any quinoa proteins (Fig. 1D).

### Comparative genomic synteny analysis

**Fig. 2:**
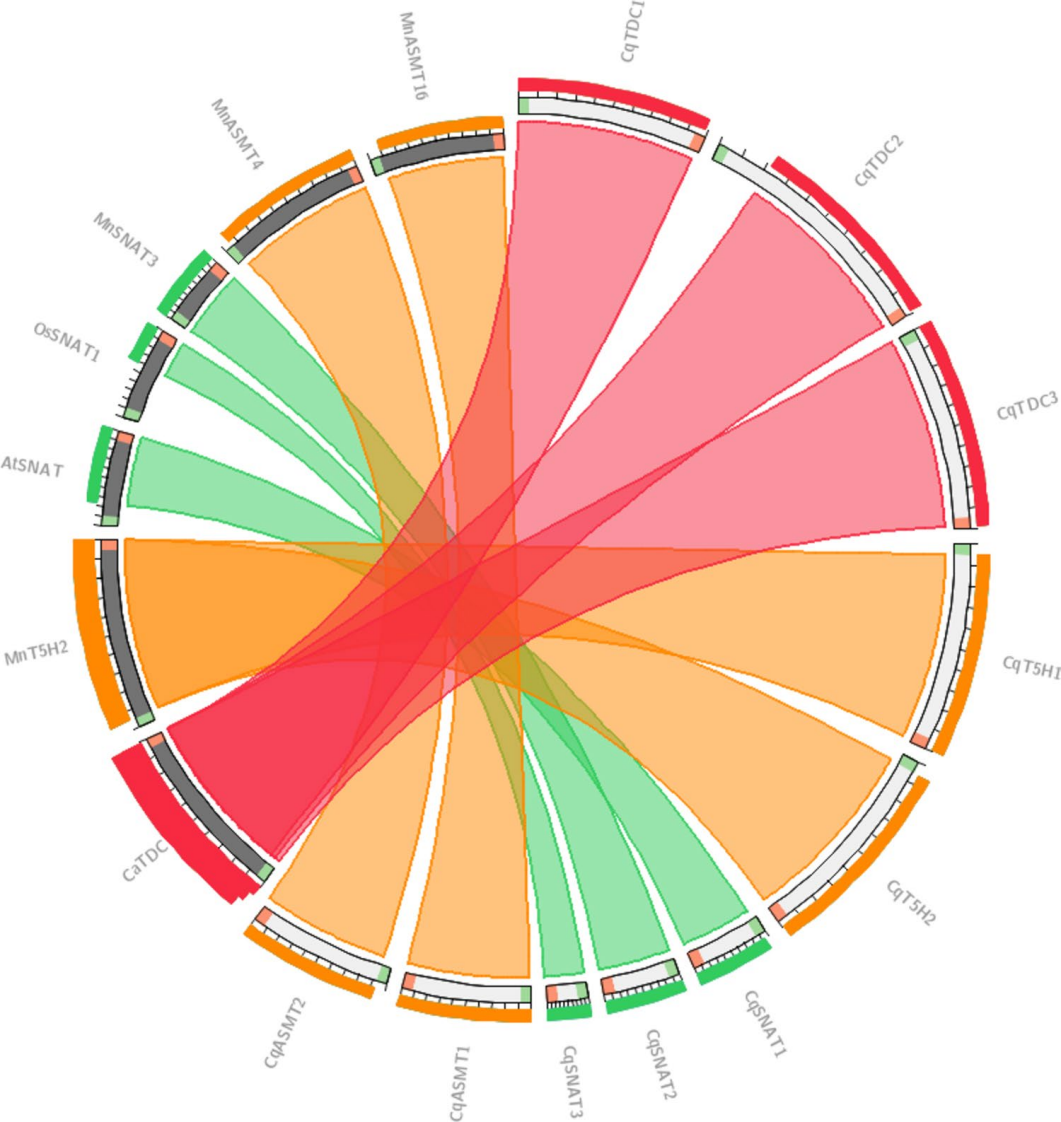
Genomic synteny between quinoa and various plant species revealing the sequence conservation levels. Different colors (red > 0.75, green ≤ 0.50 and orange ≤ 0.75) indicate evolutionary conservation levels among different genes

Genomic synteny analysis revealed sequence similarity in melatonin biosynthetic proteins of quinoa and other plant species (Fig. 2). The colors in the figure illustrate the degree of evolutionary conservation among genes. The Circoletto tool applied a 'score/max' ratio coloring scheme with green indicating scores ≤ 0.50, orange for scores ≤ 0.75, and red for scores > 0.75 (Darzentas 2010). The ribbons have been colored according to the bitscores. The analysis concluded that five melatonin biosynthetic genes from quinoa (T5H1, T5H2, SNAT2, ASMT1, and ASMT2) share a similar evolutionary origin with those from mulberry. For instance, the protein pairs CqT5H1-MnT5H2, CqASMT1-MnASMT16, and CqASMT2-MnASMT4 exhibited sequence similarities ranging from 50 to 75%. CqTDC1-3 proteins demonstrated synteny with the CaTDC from a subtropical species Chinese happy tree, exhibiting a maximum sequence similarity exceeding 75%. Three quinoa proteins, CqSNAT1-AtSNAT, CqSNAT2-MnSNAT3, and CqSNAT3-OsSNAT1, exhibited moderate-to-low similarity, ranging from 25 to 50% (green color).

### Protein domains and structure of genes

**Fig. 3:**
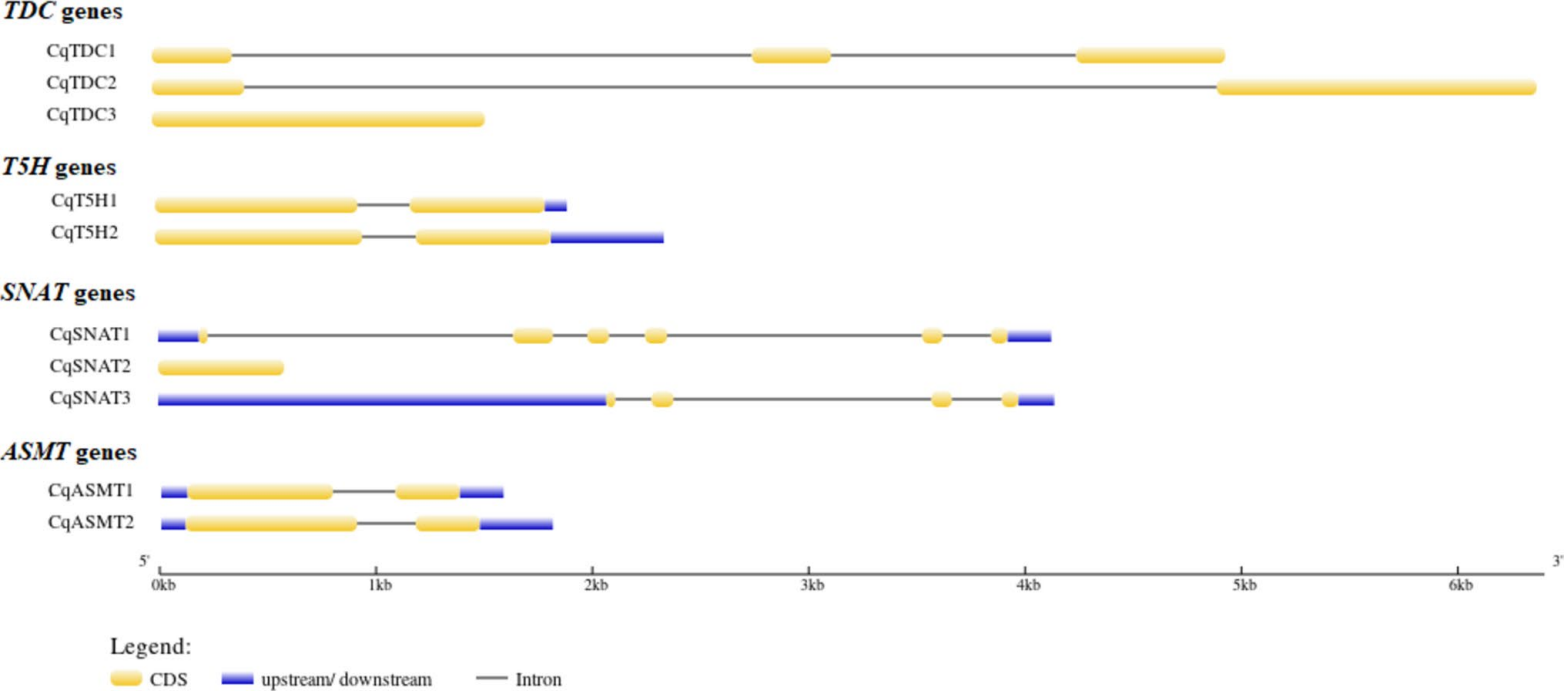
Structure analyses of the *TDC, T5H, SNAT,* and *ASMT* genes in quinoa performed in GSDS tool. Exons and introns are indicated by yellow colors and black lines, respectively

**Fig. 4:**
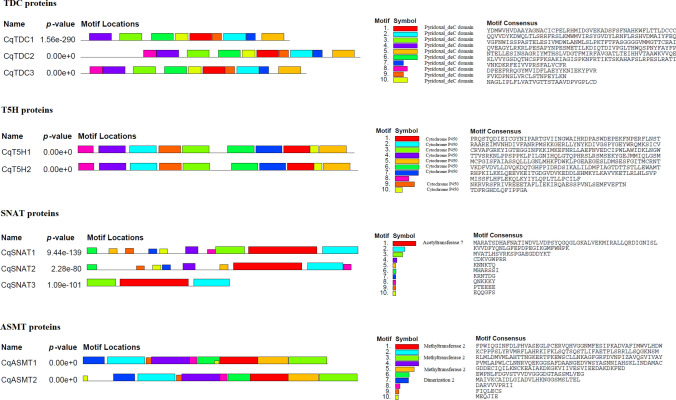
Conserved motif analyses of the TDC, T5H, SNAT, and ASMT proteins in quinoa performed in MEME suite 5.5.5 online tool. Different motifs are indicated by different colors

Figures 3 and 4 show gene structures and conserved protein domains, respectively. A comprehensive analysis was conducted on the exon and intron structures of *TDC, TSH, SNAT,* and *ASMT* genes in quinoa. *CqSNAT2* was the shortest sequence and *CqTDC2* was the longest one among all melatonin biosynthetic genes. The *CqTDC1* gene consisted of 3 exons and 2 introns, with *TDC3* being the shortest one among *TDC* genes. The *CqT5H* genes contained only 1 intron and 2 exons. The *SNAT* genes exhibited significant variations in exon and intron organizations. *CqSNAT1* was found to have 6 exons and 5 introns, whereas *CqSNAT2* gene had a single exon. Two intronless genes (*TDC3* and *SNAT2*) were detected. Both *ASMT1* and *ASMT2* genes were composed of 2 exons and 1 intron each (Fig. 3).

Using the MEME suite tool, ten conserved motifs were identified in the TDC, TSH, SNAT, and ASMT proteins (Bailey and Elkan 1994) (Fig. 4). The amino acid lengths of motifs varied from 6 to 50 for SNAT and ASMT proteins, 21 to 50 aa for TDCs, and 15 to 50 aa for T5H proteins. The motifs with the shortest amino acid lengths were found in SNAT proteins among the studied proteins. Motifs 1–10, which include the pyridoxal phosphate-dependent decarboxylase domain involved in pyridoxal phosphate binding and carbon–carbon lyase activity, were present in TDC2 and TDC3, but motif 8 was absent in TDC1 (Fig. 4). Nine p450 domains were found in T5H1-2 proteins (motifs 1, 2, 3, 4, 5, 6, 7, 9, 10). The motif 1 (acetyltransferase_7) was found in all SNAT proteins. Two ASMT proteins comprised 2 types of domains including methyltransferase_2 (motifs 1, 3, 5), and dimerization 2 (motif 7).

### Identification of *cis-*regulatory elements in promoters

A total of 1015 *cis-*elements were identified in promoter sequences of *TDC, T5H, SNAT,* and *ASMT* genes in quinoa (Table S4). The PlantCARE analysis uncovered 65 unique *cis-*acting elements. The *cis-*elements in quinoa were categorized into five functional classes: unknown or promoter-related elements (13), hormone response (10), light response (18), stress response (14), and plant development-related elements (11). There are 721 promoter-related elements (including ABRE3a, ABRE4, AT ~ TATA-box, AT-rich element, AT-rich seq, CAAT-box, CTAG-motif, Myb, MYB-like seq, Myc, TATA, TATA-box, TCA), 103 light-response elements (including AE-box, AT1-motif, ATC-motif, Box 4, chs-CMA1a, chs-CMA2a, chs-CMA2c, chs-UNIT 1 m1, G-box, GA-motif, GATA-motif, GC-motif, GT1-motif, I-box, MRE, Sp1, TCCC-motif, TCT-motif), 52 abscisic acid elements (including ABRE, MYC, MYB), 7 auxin-related elements (including AuxRR-core, TGA-element), 20 methyl jasmonate (MeJA)-related elements (including CGTCA-motif, TGACG-motif), 10 gibberellin-response elements, (including GARE-motif, P box, TATC-box), 2 salicylic acid-specific elements (including TCA-element), 9 ethylene-related elements (ERE), 134 elements related to stress-response (including ABRE, ARE, as-1, DRE1, LTR, MBS, MYB, Myb-binding site, MYB-recognition site, MYC, STRE, TC-rich repeats, W-box, WRE3, WUN-motif), and 22 elements specific to plant development (including AAGAA-motif, AC-II, CAT-box, CCAAT-box, circadian, dOCT, F-box, HD-Zip 1, O2-site, plant_AP-2-like, RY-element).

The *TDC, T5H, SNAT,* and *ASMT* genes harbored a total of 93, 58, 91, and 75 *cis-*elements, respectively (Table S5). Among them, the most abundant *cis-*elements were found in *TDC* promoters, with 46% of these elements being specific to stress response. The largest group consisted of common or promoter-related *cis-*elements, followed by the second largest group, which comprised 134 elements specific to the stress response. TATA-box (337) and CAAT-box (308) were the most abundant *cis-*elements in quinoa melatonin biosynthetic genes. Among 134 stress-responsive motifs, MYB (21), MYC (21), STRE (18), and ARE (14) were the most abundant elements with the highest frequency of 55%. The promoter of *CqTDC3* had the highest numbers of elements related to stress response. MYB and MYC are recognized as key regulators of the stress response in plants. Melatonin biosynthesis genes contained 21 MYB motifs in total, with the exception of *TDC3* and *T5H1* genes. The results revealed that nine out of ten genes in quinoa harbored MYC indicating that the melatonin biosynthesis is associated with abiotic stress response. Quinoa *SNAT* genes did not contain abscisic acid-responsive elements (ABRE). Stress-responsive elements (STRE) were found in all genes, except for *TDC1, SNAT2*, and *SNAT3*. The DRE1 and MYB-recognition site were present only in the promoters of *TDC2* and *SNAT2,* respectively. W-box elements, which serve as direct targets for WRKY transcription factors (Rinerson et al. 2015), were found in 60% of the genes involved in melatonin biosynthesis in quinoa. Wound-responsive WUN-motifs were also found in 8 genes, except for *SNAT3,* and *ASMT1*. The promoter regions of *ASMT* genes lacked TC-rich repeats. The third largest category was the light-responsive elements (103), consisting of 18 types of *cis-*elements. Among these, Box 4, G-box, and TCT-motif are the most prevalent with 37, 14, and 10 elements, respectively. The highest amounts of Box 4 were found in both *CqASMT1* (9) and *CqASMT2* (7) genes, whereas no Box4 elements were present in *CqSNAT2,* and *CqSNAT3* promoters. Among the genes analyzed, the promoter region of *CqASMT1* has the highest numbers of light-responsive elements, suggesting the potential role of quinoa *ASMT* genes in light response. The hormone-response category with 58 motifs included highest numbers of elements related to ABA, MeJA, and ethylene hormones, suggesting the roles of melatonin biosynthesis in hormonal regulation. The promoter region of *TDC3* was found to contain only one ABRE. The *CqTDC3* promoter, having a greater number of stress-response elements (mostly ARE and STRE) than other genes, is assumed to be linked to stress response instead of hormonal regulation. Auxin-related AuxRR-core was present only in the *T5H1, T5H2,* and *SNAT2* promoters. No gibberellin-response elements were identified in *ASMTs, TDC1,* and *TDC2* genes. MeJA-related elements, including CGTCA-motif and TGACG-motif, were present in the promoters of *TDC1, TDC2, T5H2, SNAT2, SNAT3, ASMT1,* and *ASMT2*. Our analysis also identified the presence of ethylene-responsive elements (ERE) in 6 promoters (*TDC2, T5H1, SNAT1-3,* and *ASMT2*) and salicylic acid-responsive TCA-elements in 2 genes, including *SNAT1,* and *ASMT1*. In addition to hormonal regulation, stress response, and light response, the PlantCARE analysis predicted the presence of *cis-*elements associated with developmental processes, such as zein metabolism (O2-site), circadian control (circadian), palisade mesophyll cells (HD-Zip1), and meristem expression (CAT-box). The lowest number of *cis-*elements (AC-II, CCAAT-box, dOCT, HD-Zip1, and plant_AP-2-like) was specific to plant development. AAGAA-motif was found in maximum abundance. The AAGAA-motif was most abundantly present, but it was only found in *TDC1-3* and *ASMT2* genes (Table S5).

### GO enrichment and PPI network of melatonin biosynthesis

**Fig. 5:**
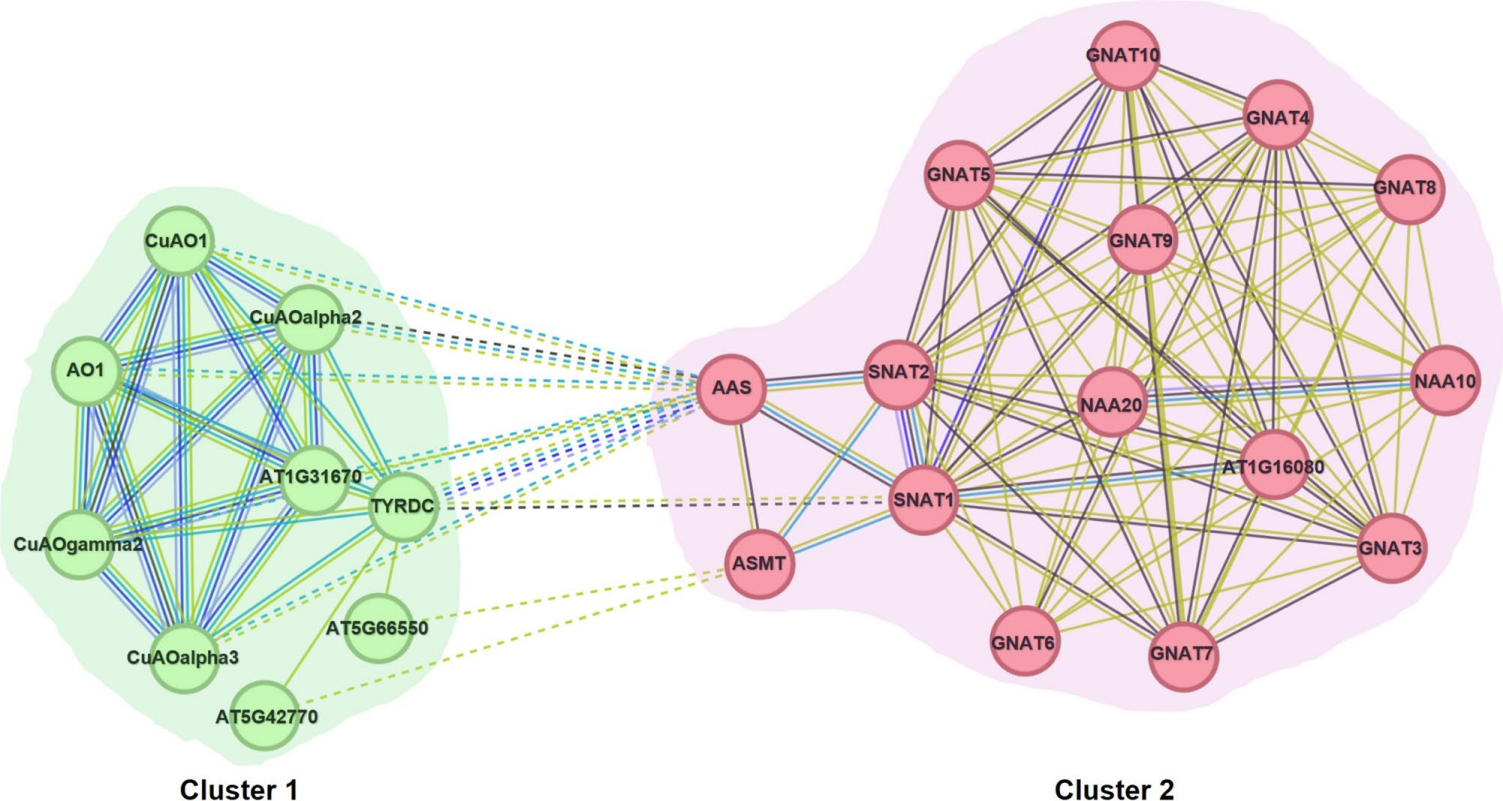
Protein–protein interaction network of melatonin biosynthesis pathway genes in *Arabidopsis thaliana*

To further investigate the functions of genes in the melatonin biosynthesis pathway and their interactions with other proteins, we constructed protein–protein interaction (PPI) networks using STRING (Fig. 5). K-means clustering grouped 9 and 15 proteins into Cluster 1 and Cluster 2, respectively. Cluster 1 included proteins involved in isoquinoline alkaloid biosynthesis, including orthologs of *TDC1* and *TDC2*. Cluster 2 consisted of proteins containing a GNAT (Gcn5-related N-acetyltransferase) domain, including orthologs of *SNAT* and *ASMT* genes. The orthologs of *T5H1* and *T5H2* did not interact with any proteins in the PPI network.

**Fig. 6:**
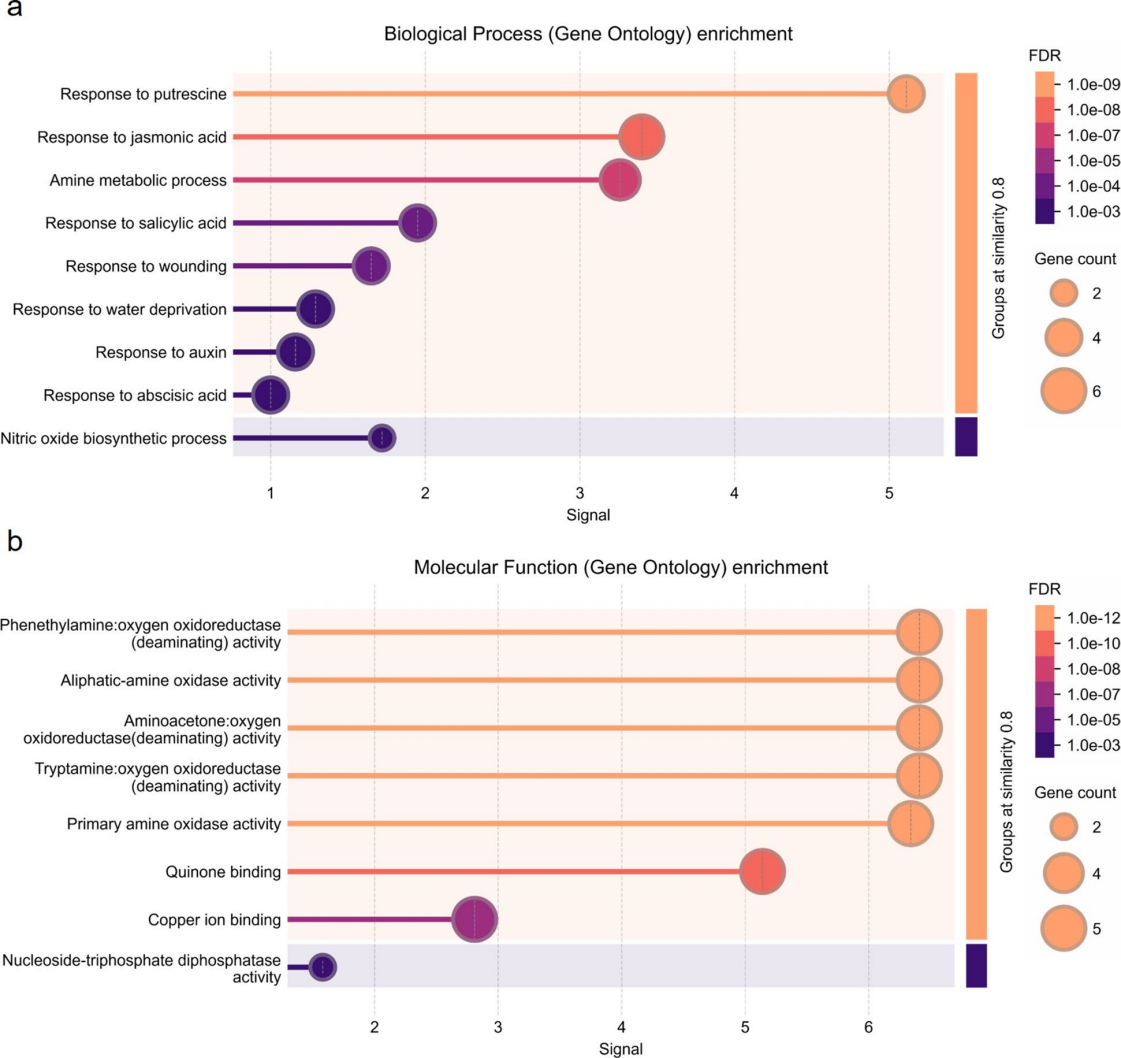
GO enrichment analysis of cluster 1 proteins: **(a)** Biological process enrichment and **(b)** molecular function enrichment

**Fig. 7:**
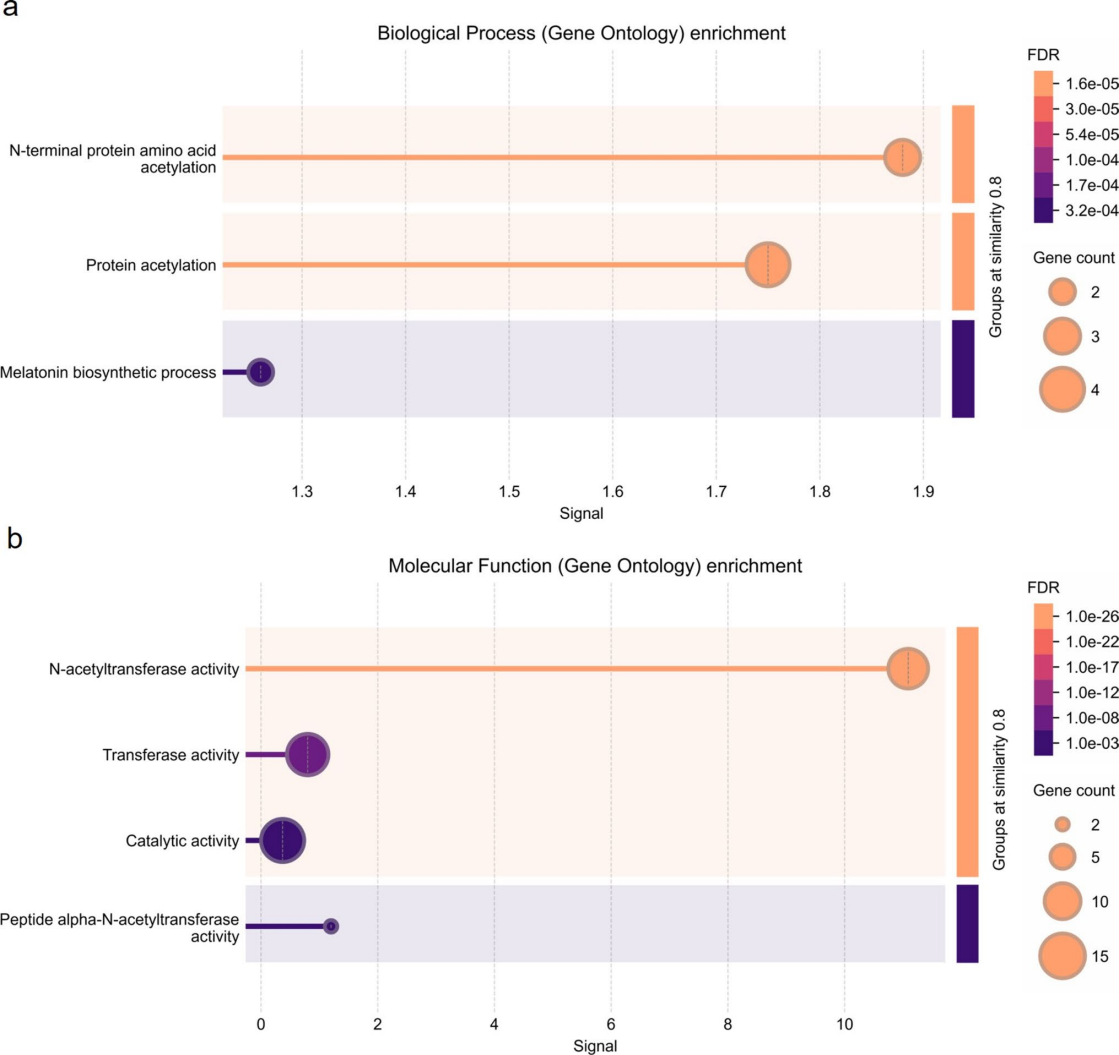
GO enrichment analysis of Cluster 2 proteins: **(a)** Biological process enrichment and **(b)** molecular function enrichment

To understand the functional roles of these clusters, we performed GO enrichment analysis for biological process and molecular function. Cluster 1 was enriched in"response to putrescine," "response to jasmonic acid," and "amine metabolic process," while Cluster 2 was enriched in "N-terminal protein amino acid acetylation" and "protein acetylation" (Fig. 6a, Fig. 7a). Taken together, GO enrichment analysis suggests that Cluster 1 is primarily associated with abiotic stress response, while Cluster 2 is linked to post-translational modifications. The enrichment of biological processes such as responses to putrescine and jasmonic acid reveals the potential involvement of these genes in stress response and metabolic regulation. Together, these results suggest that the melatonin biosynthesis pathway interacts with broader signaling and metabolic networks, playing a critical role in plant resilience to environmental challenges.

### Effects of melatonin application on growth and physiological parameters under salt and drought stress

**Fig. 8:**
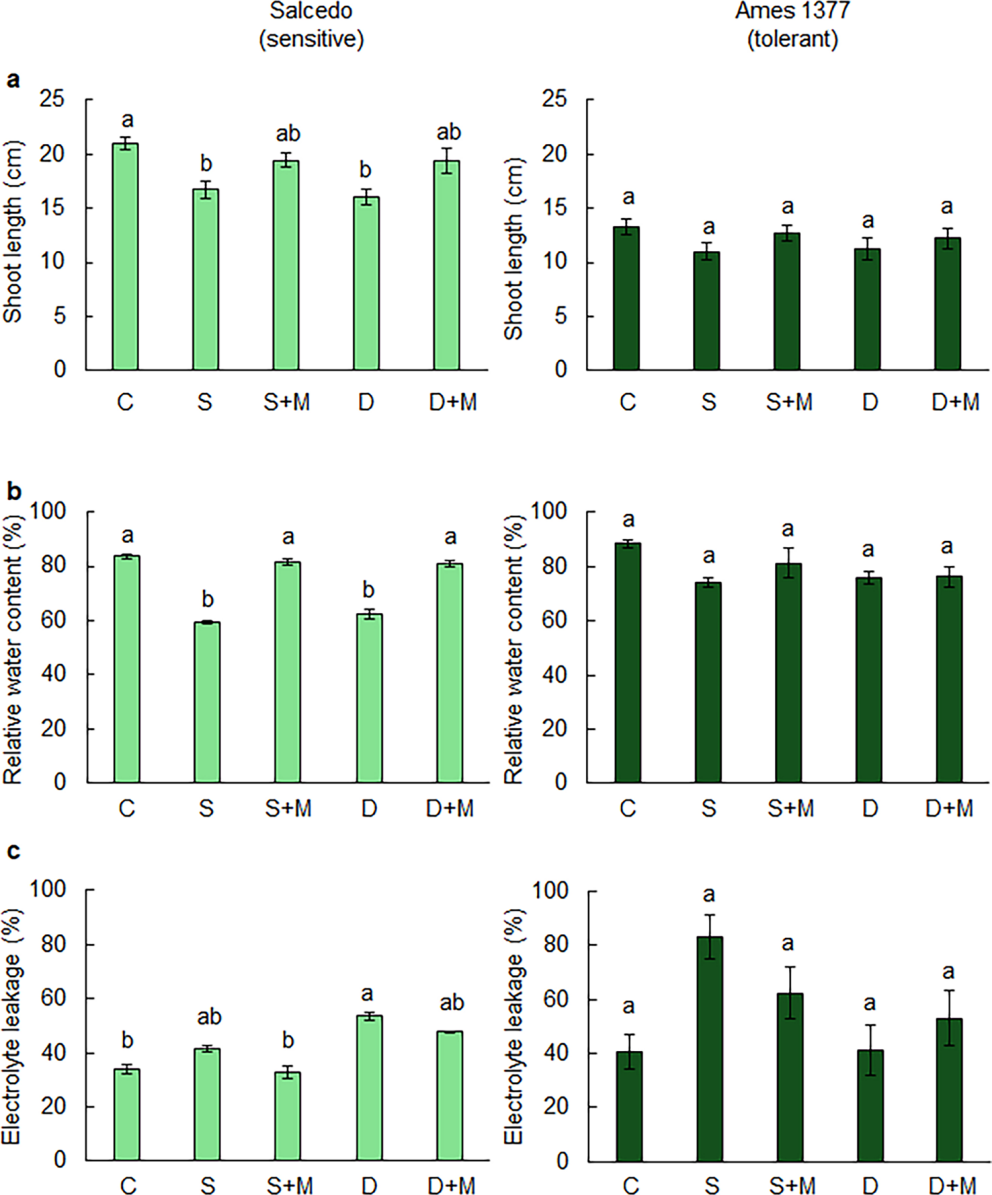
Effects of melatonin application under salt and drought stress on shoot length, relative water content, and electrolyte leakage. (**a**) Shoot length. (**b**) Relative water content (RWC). (**c**) Electrolyte leakage. 20-day-old Salcedo (sensitive) and Ames 13,777 (tolerant) quinoa genotypes were exposed to salinity (300 mM NaCl), drought (40% FC), salinity (300 mM NaCl) + melatonin (70 µM), or drought (40% FC) + melatonin (70 µM) for 15 days. Letters indicate statistical differences among the control and stress groups determined by ANOVA followed by Tukey post hoc test (*p* < 0.05) within each genotype. C: Control, S: Salt, S + M: Salt + Melatonin, D: Drought, D + M: Drought + Melatonin

To understand how melatonin treatment affects the growth and physiological responses of quinoa genotypes with contrasting stress tolerance levels under drought and salt stresses, we exposed them to stress conditions for 15 days, with or without melatonin treatment. Salt stress significantly decreased shoot length in sensitive Salcedo by 20.3% relative to the control (*p* = 0.0016) (Fig. 8a). However, applying melatonin to salt-stressed plants increased shoot length significantly by 16% compared to the salt-only treatment (*p* = 0.0461). Similarly, drought reduced shoot length in Salcedo by 23.7% (*p* = 0.0004) relative to the control, whereas melatonin application resulted in a 21.3% increase compared to drought-only plants (*p* = 0.0578). In contrast, salt and drought treatments caused only slight, statistically non-significant decreases in shoot length in the tolerant Ames 1377 genotype.

The RWC in Salcedo declined significantly under both salt (29.1%, *p* < 0.05) and drought (25.4%, *p* < 0.05) compared with the control (Fig. 8b). Ames 1377 also exhibited decreases in RWC under salt (16.3%, *p* < 0.05) and drought (14.2%, *p* < 0.05) conditions, although melatonin treatment did not significantly improve RWC in this genotype. In Salcedo, however, the application of 70 µM melatonin under salt stress increased RWC by 37.6% (*p* < 0.05) and under drought stress by 29.3% (*p* < 0.05), both relative to the corresponding stress condition.

In Salcedo, salt stress resulted in a 21.7% increase in electrolyte leakage compared to the control (*p* < 0.05), whereas melatonin application led to a 20.6% reduction (*p* = 0.0253) (Fig. 8c). Similarly, drought treatment significantly increased electrolyte leakage by 56.8% (*p* < 0.05), and melatonin application under drought lowered it by 11.9% compared to drought (*p* = 0.585), bringing it back to the control levels. Electrolyte leakage doubled (*p* < 0.05) only under salt stress in Ames 1377. Although melatonin treatment slightly reduced electrolyte leakage in salt-stressed Ames 1377 compared to the stress condion, the reduction was not statistically significant (*p* = 0.275). Taken together, these results suggest that melatonin treatment effectively mitigates the adverse effects of salt and drought stresses on shoot length, RWC, and electrolyte leakage in the sensitive Salcedo genotype. However, its protective role appears limited or non-significant in the stress-tolerant Ames 1377 genotype, indicating that the efficacy of melatonin may depend on the inherent stress tolerance mechanisms of the genotype.

Ames 1377 (tolerant) and Salcedo (sensitive) differed significantly across all measured traits, shoot length (*p* < 0.001), RWC (*p* = 0.001), and electrolyte leakage (*p* < 0.001), and both salt and drought treatments exerted a significant main effect on each parameter (*p* < 0.001) (Table S6). A significant genotype × treatment interaction was detected for shoot length and RWC (*p* < 0.05), indicating that melatonin’s efficacy varied with genotype, whereas no interaction was observed for electrolyte leakage (*p* = 0.20), suggesting a uniform stress response across genotypes.

### Gene expression profiles under salt and drought

**Fig. 9:**
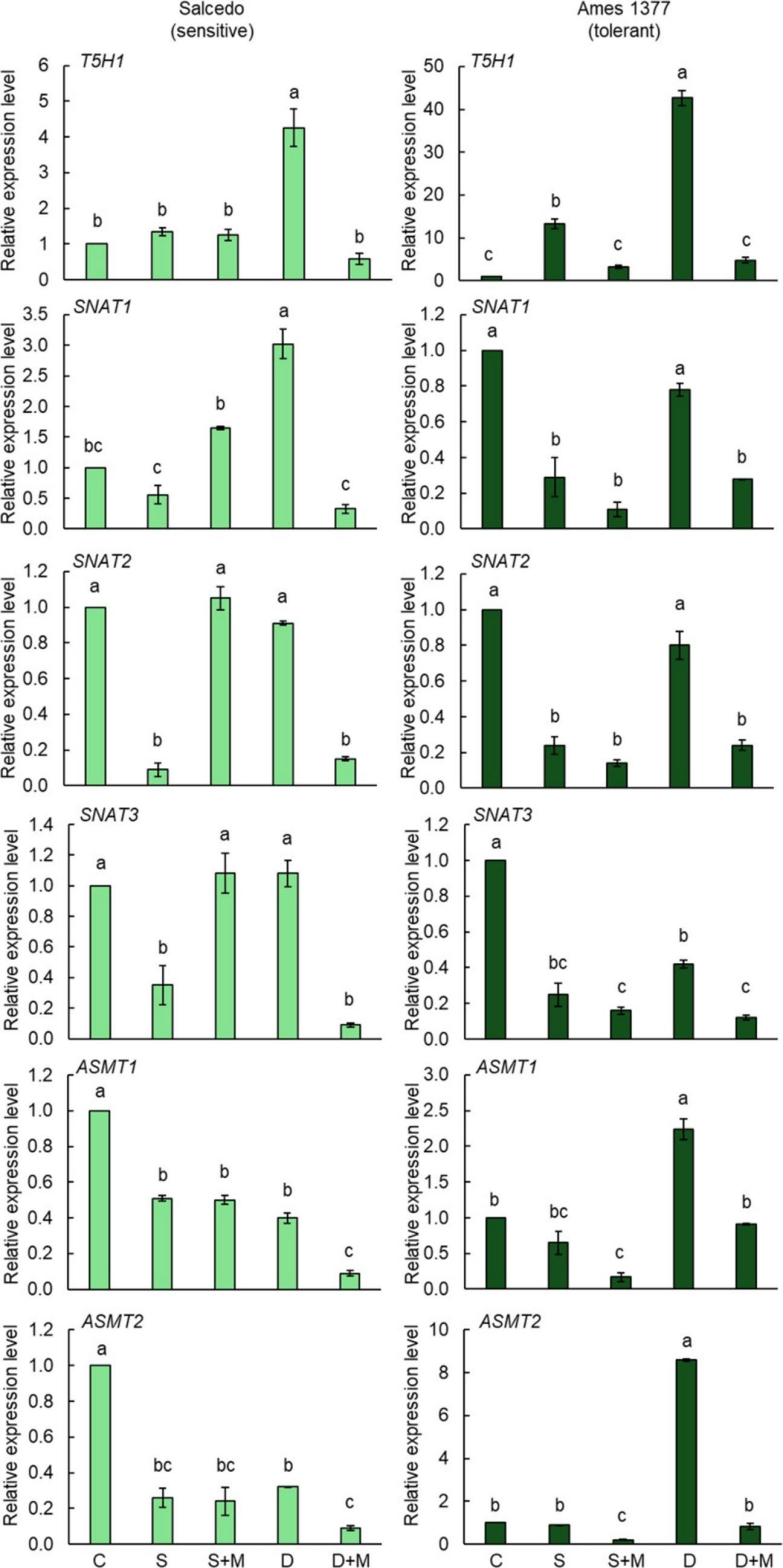
Gene expression levels of melatonin biosynthesis genes in the **leaves** of Salcedo (sensitive) and Ames 1377 (stress-tolerant) quinoa genotypes under salt and drought stresses with or without melatonin treatment. 20-day-old Salcedo (sensitive) and Ames 13,777 (tolerant) quinoa genotypes were exposed to salinity (300 mM NaCl), drought (40% FC), salinity (300 mM NaCl) + melatonin (70 µM), or drought (40% FC) + melatonin (70 µM) for 15 days. Letters indicate statistical differences among the control and stress groups determined by ANOVA followed by Tukey post hoc test (*p* < 0.05) within each genotype. C: Control, S: Salt, S + M: Salt + Melatonin, D: Drought, D + M: Drought + Melatonin

**Fig. 10:**
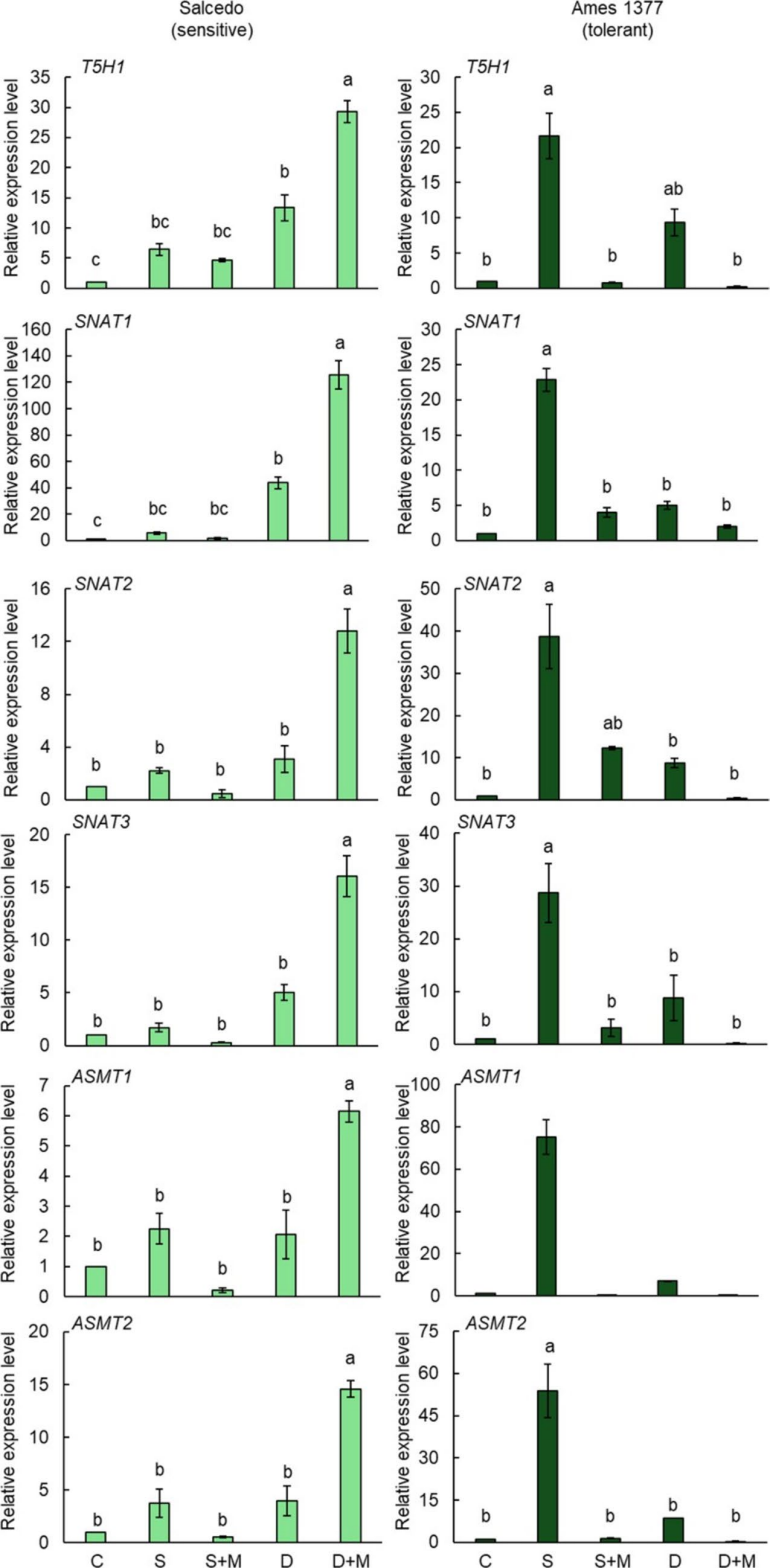
Gene expression levels of melatonin biosynthesis genes in the **roots** of Salcedo (sensitive) and Ames 1377 (stress-tolerant) quinoa genotypes under salt and drought stresses with or without melatonin treatment. 20-day-old Salcedo (sensitive) and Ames 13,777 (tolerant) quinoa genotypes were exposed to salinity (300 mM NaCl), drought (40% FC), salinity (300 mM NaCl) + melatonin (70 µM), or drought (40% FC) + melatonin (70 µM) for 15 days. Letters indicate statistical differences among the control and stress groups determined by ANOVA followed by Tukey post hoc test (*p* < 0.05) within each genotype. C: Control, S: Salt, S + M: Salt + Melatonin, D: Drought, D + M: Drought + Melatonin

Figures 9 and 10 present the expression patterns of 6 melatonin biosynthesis genes in leaves and roots under salt and drought stress, with or without melatonin application. We performed qRT-PCR analysis of all genes in the control and drought- or salt-treated quinoa groups. Among the analyzed genes, we found differences between control and stress-treated groups only in *T5H1, SNAT1, SNAT2, SNAT3, ASMT1,* and *ASMT2* genes. The remaining four genes, including *T5H2, TDC1, TDC2,* and *TDC3,* did not exhibit differences in their expression abundances. In Salcedo, salt stress caused a modest 34% increase in *T5H1* expression in the leaves, and melatonin application did not significantly alter this (Fig. 9). Under drought conditions, *T5H1* expression in the leaves was strongly upregulated by 3.25-fold, while melatonin supplementation suppressed it by 86.1%. *T5H1* expression in the roots increased dramatically by 5.42-fold under salt stress, which melatonin reduced by 26.7% (Fig. 10). Drought stress caused a 12.4-fold increase in *T5H1* expression in the roots, followed by an additional 1.19-fold enhancement with melatonin treatment. *SNAT1* expression in the leaves was suppressed by 44% under salt stress, while melatonin treatment enhanced it by 1.95-fold (Fig. 9). Under drought stress, *SNAT1* expression in the leaves doubled significantly, but melatonin supplementation downregulated it by 89% compared to drought-only conditions. *SNAT1* expression in the roots increased 4.6-fold under salt stress, which was mitigated by 70% with melatonin application. Drought stress led to a striking 42.86-fold increase in *SNAT1* expression in the roots, and melatonin further enhanced this by an additional 1.86-fold (Fig. 10). *SNAT2* expression in the leaves was markedly reduced by 91% under salt stress and slightly downregulated by 9% under drought conditions. When melatonin was applied during drought, *SNAT2* expression in the leaves was suppressed by additional 83.5%, whereas under salt stress, melatonin caused a dramatic 10.66-fold enhancement (Fig. 9). *SNAT2* expression in the roots was elevated by 1.21-fold under salt stress, and melatonin suppressed it further by 79.6% (Fig. 10). Although drought stress increased *SNAT2* expression in the roots, the change was not statistically significant. Interestingly, melatonin treatment under drought resulted in a remarkable 3.14-fold increase in *SNAT2* expression in the roots compared to drought stress alone. For *SNAT3* expression, salt stress reduced levels in the leaves by 65%, while melatonin significantly induced a 2.08-fold increase. Drought stress did not significantly affect *SNAT3* expression in the leaves; however, melatonin supplementation suppressed it by 83% under drought conditions (Fig. 9). *SNAT3* expression in the roots increased by 70% under salt stress, but melatonin reversed this effect with a 71% reduction (Fig. 10). Under drought, *SNAT3* expression in the roots increased significantly by 4-fold, and melatonin further enhanced this by 2.18-fold. *ASMT1* expression in the leaves declined by 49% under salt stress, and melatonin treatment did not alter it further (Fig. 9). Drought stress upregulated *ASMT1* expression in the leaves by 60%, but melatonin supplementation caused a 77.8% reduction. *ASMT1* expression in the roots increased by 1.25-fold under salt stress, which melatonin suppressed by 50% (Fig. 10). Drought stress did not significantly affect *ASMT1* expression in the roots; however, melatonin treatment induced a 2-fold increase compared to drought conditions alone. For *ASMT2* expression, salt stress downregulated it in the leaves by 74%, and melatonin treatment had no significant effect (Fig. 9). Under drought stress, *ASMT2* expression in the leaves was suppressed by 68%, and melatonin caused an additional reduction of 85.07%. *ASMT2* expression in the roots increased 2.75-fold under salt stress, which melatonin mitigated by 44% (Fig. 10). Although drought stress slightly enhanced *ASMT2* expression in the roots, the change was not statistically significant. However, melatonin supplementation triggered a 2.66-fold increase in *ASMT2* expression in the roots, leading to a net 13.59-fold rise compared to the control.

In the Ames 1377, salt stress caused 12.3-fold increase in *T5H1* expression in the leaves, while melatonin mitigated this increase by 96.3% (Fig. 9). Drought stress induced a dramatic 41.68-fold up-regulation in *T5H1* expression in the leaves, and melatonin suppressed it by 88.7%. *T5H1* expression in the roots increased 20.7 times under salt stress, which melatonin reduced by 96% (Fig. 10). Drought caused an 8.4-fold increase in *T5H1* expression in the roots, followed by an 88.3% reduction with melatonin application. The *SNAT1* expression in the leaves was significantly downregulated under both salt and drought stress (Fig. 9). Salt stress suppressed *SNAT1* expression in the leaves by 71%, and melatonin treatment reduced it further by 62.1%. Drought stress led to a 22% reduction in *SNAT1* expression in the leaves, with melatonin causing an additional 64.1% suppression. *SNAT1* expression in the roots increased by 21.85-fold under salt stress, which melatonin application reduced by 82.3% (Fig. 10). Drought stress enhanced *SNAT1* expression in the roots by 4-fold, but melatonin supplementation mitigated this increase by 60%. *SNAT2* expression in the leaves was significantly suppressed by 76% under salt stress, with melatonin causing an additional 41.7% reduction (Fig. 9). Drought stress downregulated *SNAT2* expression in the leaves by 20%, and melatonin further suppressed it by 70%. *SNAT2* expression in the roots increased by 37.7 times under salt stress, which melatonin treatment reduced by 68.2% (Fig. 10). Drought stress upregulated *SNAT2* expression in the roots by 8-fold, while melatonin suppressed this change by 95%. Salt stress caused a 75% reduction in *SNAT3* expression in the leaves, and melatonin had no further effect (Fig. 9). Drought stress downregulated *SNAT3* expression in the leaves by 58%, and melatonin application caused an additional 71% suppression. *SNAT3* expression in the roots increased dramatically by 27.7 times under salt stress and 7.8 times under drought stress (Fig. 10). Melatonin treatment significantly reduced *SNAT3* expression in the roots by 89.1% under salt stress and 71.4% under drought stress. *ASMT1* expression in the leaves remained unchanged under salt stress, but melatonin reduced it by 83% compared to the control (Fig. 9). Drought stress upregulated *ASMT1* expression in the leaves by 1.24-fold, while melatonin caused a 59% suppression. *ASMT1* expression in the roots increased by 74.4 times under salt stress, which melatonin nearly completely reversed, reducing it by 99.4% (Fig. 7). Drought stress enhanced *ASMT1* expression in the roots by 6-fold, and melatonin mitigated this increase by 51%. For *ASMT2* expression in the leaves, salt stress caused a slight 12% reduction, and melatonin suppressed it further by 77.3% (Fig. 9). Drought stress strongly upregulated *ASMT2* expression in the leaves by 7.6 times, but melatonin treatment caused a dramatic 90.5% reduction. *ASMT2* expression in the roots increased by 52.8 times under salt stress, which melatonin suppressed by 97.3% (Fig. 10). Similarly, drought stress upregulated *ASMT2* expression in the roots by 7.5-fold, and melatonin reduced it by 96%.

*SNAT1* shows strong genotype × stress interactions in both roots and leaves, suggesting its key role in differential stress adaptation. *SNAT2* functions as a core stress responder in roots but displays genotype-dependent leaf expression, while *SNAT3* acts as a universal stress responder in roots but shows dramatic genotype-specific leaf responses. *ASMT1* presents a striking contrast—displaying genetic regulation in roots but complete conservation in leaves. *ASMT2* emerges as the most consistently genotype × environment responsive gene across both tissues, indicating its central role in stress-induced melatonin production. *T5H1* shows the strongest genetic effects in roots but more moderate leaf responses, highlighting tissue-specific serotonin pathway regulation. These findings demonstrate that genes in the melatonin biosynthesis pathway showed differential responses between roots and leaves under salt and drought conditions in Salcedo and Ames 1377. This shows the importance of both genotype-specific and tissue-specific adaptations to these stressors. Additionally, exogenous melatonin application revealed critical differences in how these genes are regulated in response to stress.

## Discussion

Melatonin could serve as a valuable tool for developing abiotic stress-tolerant crops due to its involvement in the regulation of ROS production, nitrogen metabolism, antioxidant defense, H^+^-ATPase function, and stress response (Ahmad et al. 2024; Khan 2023; Li et al. 2022). The amphiphilic nature of melatonin allows it to be present in all cellular compartments, including the membranes, cytoplasm, nucleus, and mitochondria, where it plays a role in ROS scavenging (Reiter et al. 2013). Exogenous melatonin application has been reported to delay abiotic stress-triggered leaf senescence and cell death in rice (Liang et al. 2015), cabbage (Tan et al. 2019), and apple (Wang et al. 2012). Melatonin prevented the decline in chlorophyll content and maximum potential efficiency of photosystem II, and decreased the expression of a chlorphyll degradation gene, *pheide a oxygenase (PAO)*, and *senescence-associated gene 12 (SAG12)* (Wang et al. 2012). Additionally, melatonin regulates several physiological processes, including seed germination (Chen et al. 2020), flowering (Byeon and Back 2014), and circadian rhythm (Chang et al. 2021). These findings demonstrate the role of melatonin as a multifunctional molecule (Priti et al. 2024). Exploring the melatonin biosynthesis pathway in different plants could deepen our understanding of its regulatory roles and highlight its potential for improving stress tolerance and antioxidant capacity in crops.

In this study, we identified 3 TDCs, 2 TSHs, 3 SNATs, and 2 ASMTs in quinoa genome for the first time, which could be important in elucidating the underlying mechanisms of abiotic stress tolerance in quinoa. In a recent study, 7 and 9 *TDC* genes, 2 and 1 *SNAT* genes, and 19 and 28 *ASMT* genes were identified in rice and sorghum (*Sorghum bicolor*), respectively (Bhowal et al. 2021). *Arabidopsis* and tomato had 2 and 5 *TDC* genes, 1 and 2 *SNAT* genes, and 17 and 11 *ASMT* genes. In addition, only one *T5H* gene was present in *Arabidopsis*, sorghum and rice, but six isoforms of *TSH* gene were identified in tomato (Bhowal et al. 2021). In carnation (*Dianthus caryophyllus* L.), two TDC, two T5H, one SNAT, and five ASMT members were identified (Priti et al. 2024). Recently, in tomato, 3 *TDC* genes and a single *T5H* gene were identified by homology-based screening and transient expression in tobacco (*Nicotiana benthamiana)* (Commisso et al. 2022). TDC is a first enzyme in melatonin biosynthesis that is a type II pyridoxal-5′-phosphate-dependent decarboxylase (Zhou et al. 2020). The SNAT enzymes, which use acetyl-coenzyme A (CoA) as a cofactor (Byeon et al. 2013), are poorly conserved in *Arabidopsis,* rice, and mulberry (Zheng et al. 2021). In *Arabidopsis*, tomato, mulberry*,* bell pepper *(Capsicum annuum)*, and sorghum, there are 17, 14, 20, 16, and 28 *ASMT* genes, respectively (Zheng et al. 2021; Liu et al. 2017; Bhowal et al. 2021; Pan et al. 2019). By contrary, only two ASMT proteins were identified in quinoa, which could be related to gene loss during evolution. In general, protein motifs with similar compositions may be unique to a family or group, which is an indication of similar biological functions. Like quinoa proteins, the TDCs had pyridoxal-dependent decarboxylase domain, the T5Hs had cytochrome P450, the SNAT proteins contained the Acetyltransf_1 domain, and ASMTs had the O-methyltransferase conserved domain (Priti et al. 2024). Eleven motifs were present in the seven OsTDC proteins belonging to aldehyde dehydrogenase (DUF674 and AAT_I) superfamily (Wang et al. 2025). In carnation*,* the ClustalW analysis revealed the presence of a conserved domain HKWL in TDCs, cysteine heme–iron ligand domain- FGAGRRICPA in T5Hs, acetyl-CoA binding motif-PSYQGQLGKA in SNAT, and adenosine-L-methionine -DVGG and DLF in ASMTs (Priti et al. 2024).

In quinoa, we determined physical and chemical properties of the genes/proteins and their exon–intron structures. Quinoa T5H proteins exhibited molecular weights of 59 and 60 kDa, while the molecular weights of TDC proteins were respectively found 52.71 and 57 kDa. In rice, Wang et al. (2025) identified 9 *OsTDC* genes, and their amino acid lengths were 514–526 aa, the MWs ranged from 55.7 to 56.5 kDa, similar to quinoa TDC1 and TDC3. In a recent work, the molecular weights of T5H in carnation were 48 and 57 kDa, while TDC1 and TDC2 had molecular weights of 53 and 56 kDa, respectively (Priti et al. 2024). The protein lenghts of T5Hs in *Arabidopsis*, rice, tomato, and sorghum ranged from 338 to 523 aa, and their pI values varied from 5.9 to 8.5 (Bhowal et al. 2021). The *TDC3* gene in quinoa was found to have no introns, but *TDC1* and *TDC2* had 2 and 1 introns, respectively. Consistently, *TDC* gene in peony consisted of 1849 bp with no intron, encoding a 502 aa protein with a MW of 56 kDa (Zhao et al. 2019). Eight rice *TDC* genes were found to contain 1–2 exons (Wang et al. 2025). The *CqT5H* and *CqASMT* genes had only 1 intron and 2 exons. The exon/intron organizations of melatonin biosynthesis genes in carnation showed 3–12 exons in *TDCs*, 2–3 exons in *T5Hs*, 8 exons in *SNAT*, and 2–5 exons in *ASMTs* (Priti et al. 2024). Intron abundance is linked to plant evolution, and genes with less introns lead to an efficient transcription under different environmental conditions (Schmitz-Linneweber et al. 2015). Among melatonin biosynthetic enzymes, the shortest proteins were SNATs in quinoa (105, 194, 199 aa), *Arabidopsis*, rice, tomato, and sorghum (191–258 aa) (Bhowal et al. 2021). However, alternative splicing was more likely in *CqSNAT* genes because of their higher numbers of introns, indicating functional variations in their proteins. In a recent work, carnation SNAT showed the lowest MW of 27.82 kDa, and 247 aa in length. ASMT proteins in carnation ranged from 246 to 1089 amino acids (Priti et al. 2024), while quinoa ASMT1 and ASMT2 were found to be 323 and 363 amino acids, respectively.

Using TDC, T5H, SNAT, and ASMT proteins from various plants, a phylogenetic tree was generated to determine evolutionary relationships between proteins of quinoa and other plants. CqTDC2 and CqTDC3 were related to OsTDC and AtTDC, while CqTDC1 was located at the different clade. Nine *TDC* genes in rice were found to divide into two subfamilies, DUF674 (2 rice genes) and AAT_I (7 genes) (Wang et al. 2025). The *TDC* genes in different plants share conserved gene family organization, functions, and evolutionary relationships (Zheng et al. 2021; Kolodziejczyk and Kazmierczak 2024; Wang et al. 2025). Tomato TDC1 and TDC2 showed a close relationship with TDCs from other solanaceous species, while SlTDC3 was found to be related to PepTDC2 and OsTDC2 (Commisso et al. 2022). The *TDC, T5H, SNAT,* and *ASMT* genes in model plants and crops were phylogenetically divided into five groups (I–IV) in carnation (Priti et al. 2024). Nine T5H proteins in distinct plant species exhibited a close relationship with CqT5Hs. In a previous work, the MnT5H2 was identified within the same clade as MdT5H1, while cotton T5H (GaT5H) and MnT5H6 were found to cluster together (Zheng et al. 2021). Here, MnSNAT1 and AtSNAT were found at the same clade with CqSNAT1 and CqSNAT3. The SNATs of mulberry were clustered into two branches, the MnSNAT6 existed at the same clade with AtSNAT and OsSNAT (Zheng et al. 2021). Tomato and mulberry ASMTs were classified into 3 groups. The MdASMT, OsASMT1-3, and MnASMTs were in class I, while AtASMT and MnASMT3-16–18 were present in class II (Zheng et al. 2021). In the present work, CqASMT1-2, AtASMT, and MnASMT4-16–18 clustered together, which belong to class II ASMTs.

In this study, we found the cytosolic and cytoskeleton localizations for TDC proteins, chloroplastic for T5Hs, chloroplastic, mitochondrial and cytosolic for SNATs, and cytosolic for ASMTs based on WoLF PSORT. In quinoa, four proteins—TDC1, SNAT3, ASMT1, and ASMT2—are predicted to be localized in the cytosol, suggesting that these enzymes may play roles in signal perception and transduction. The WoLF PSORT prediction tool showed nuclear and cytoplasmic localization of TDCs, endoplasmic reticulum and chloroplast location of T5Hs, chloroplastic localization in SNAT and cytoplasmic location of ASMTs in carnation (Priti et al. 2024). The subcellular localization of peony *TDC*-encoding protein was found in the cytoplasm (Zhao et al. 2019). Two TDCs (CsTDC1 and CsTDC2) in cucumber were experimentally found to be localized in the cytoplasm and plasma membrane, respectively (Zhang et al. 2024). Based on cello-life, we also determined plasma membrane localization of three TDC proteins. All SNATs from *Arabidopsis*, tomato, sorghum, and rice were assumed to be localized in chloroplast (Bhowal et al. 2021). In our case, chloroplastic, mitochondrial, and cytosolic localizations of CqSNAT1, CqSNAT2, and CqSNAT3 proteins suggest functional variation. Based on WoLF PSORT and cello-life tools, the ASMTs in distinct plants, including quinoa, salt-tolerant apple, *Arabidopsis*, rice, tomato, and sorghum, were predicted to be localized in the cytoplasm (Bhowal et al. 2021; Zuo et al. 2014). In contrast, OsASMT5/11/18/19, AtASMT6, and AtASMT15 proteins were localized in cytoskeleton and nucleus (Bhowal et al. 2021). In summary, the bioinformatics analyses highlight the different evolutionary relationships and subcellular localizations of melatonin biosynthesis proteins in quinoa.

Our PPI network and GO enrichment analysis revealed that Cluster 1 genes are associated with biological processes, such as “response to putrescine,” “response to jasmonic acid,” “response to salicylic acid,” and “amine metabolic process.” Cluster 2 genes are enriched in processes related to"N-terminal protein amino acid acetylation" and “protein acetylation” which is a key regulatory mechanism that modulates gene expression and stress responses in plants (Wang et al. 2024). Putrescine, a polyamine involved in plant stress responses, is crucial for maintaining cellular homeostasis under abiotic stress (Arnao and Hernández-Ruiz 2019). Previous studies showed that putrescine and melatonin interact to regulate oxidative stress and enhance stress tolerance by modulating polyamine metabolism (Zhao et al. 2017). Given the enrichment of Cluster 1 genes in putrescine response, it is possible that Cluster 1 genes contribute to polyamine-mediated stress adaptation, potentially functioning together with melatonin to enhance tolerance of plants under abiotic stress. In addition, Cluster 1 genes are linked to jasmonic acid (JA) and salicylic acid (SA) pathways, which are essential for plant defense and stress response. Melatonin is known to show overlapping functions with JA and SA in modulating abiotic stress response (Arnao and Hernández-Ruiz 2019). The presence of protein acetylation-related genes in Cluster 2 suggests that melatonin-mediated stress tolerance may involve the regulation of N-terminal protein acetylation.

In eukaryote genes, *cis-*elements and gene expression patterns are important for stress tolerance (Schmitz et al. 2022). In this study, we found five types of *cis-*elements related to promoters, stress response, hormone response, light response, and plant development. Besides promoter-specific *cis-*elements, the largest groups were stress- and light-response elements, including 134 and 103 members, respectively. The predominant *cis-*elements in *CqTDC* genes were linked to stress response similar to those found in rice *TDC* promoters (Wang et al. 2025), suggesting the crucial role of the proteins encoded by the *TDC* genes in stress tolerance. Moreover, promoter analysis exhibited that 11 types of *cis-*elements related to light response were found in the *TDC* promoters of cucumber (Zhang et al. 2024). However, no salicylic acid (SA)- and auxin-related motifs have been identified in quinoa * TDC*s in contrast to rice *TDCs*. The *cis-*elements in quinoa *T5H* promoters were mostly associated with stress response, while those in *SNAT* promoters were largely related to stress response and hormonal regulation. The *CqASMT* genes were found to contain highest numbers of *cis-*elements related to stress response and light response. Myeloblastosis (MYB) and Myelocytomatosis (MYC) families are among the largest transcription factors in plants, playing key roles in seed and flower development, regulation of primary and secondary metabolism, and abiotic or biotic stress response (Li et al. 2015, 2024). In the present study, stress-response-specific elements, such as MYB, MYC, STRE, and ARE, were found in the promoters of melatonin biosynthetic genes. Among them, MYB and MYC were the most abundant ones, suggesting the involvement of quinoa genes in abiotic stress response. Similarly, four distinct *cis-*element groups containing development, hormone response, stress response, and light response were predicted in the promoters of *TDC, T5H, SNAT, *and *ASMT* of carnation, with stress-specific ones being the most abundant (Priti et al. 2024). More than 80% of the *cis-*elements were related to stress response in all melatonin biosynthesis genes of carnation (Priti et al. 2024). A higher abundance of ABRE elements was identified in the promoters of *CqTDC2* and *CqT5H2*, but no ABRE elements were detected in *CqSNAT* promoters. Among genes, *CqTDC1, CqTDC2, CqT5H1, CqSNAT2-3,* and *CqASMT1* were found to be included W-box (TTGACC), which is involved in pathogen-induced activity, and is a binding site for WRKY transcription factors (Eulgem et al. 2000). In both cucumber and quinoa, there were *cis-*elements specific to hormones, such as ABA, gibberellin, MeJA, and ethylene (Zhang et al. 2024). These findings suggest that melatonin biosynthesis genes in quinoa may be regulated by stress factors, light, and hormones.

**Fig. 11:**
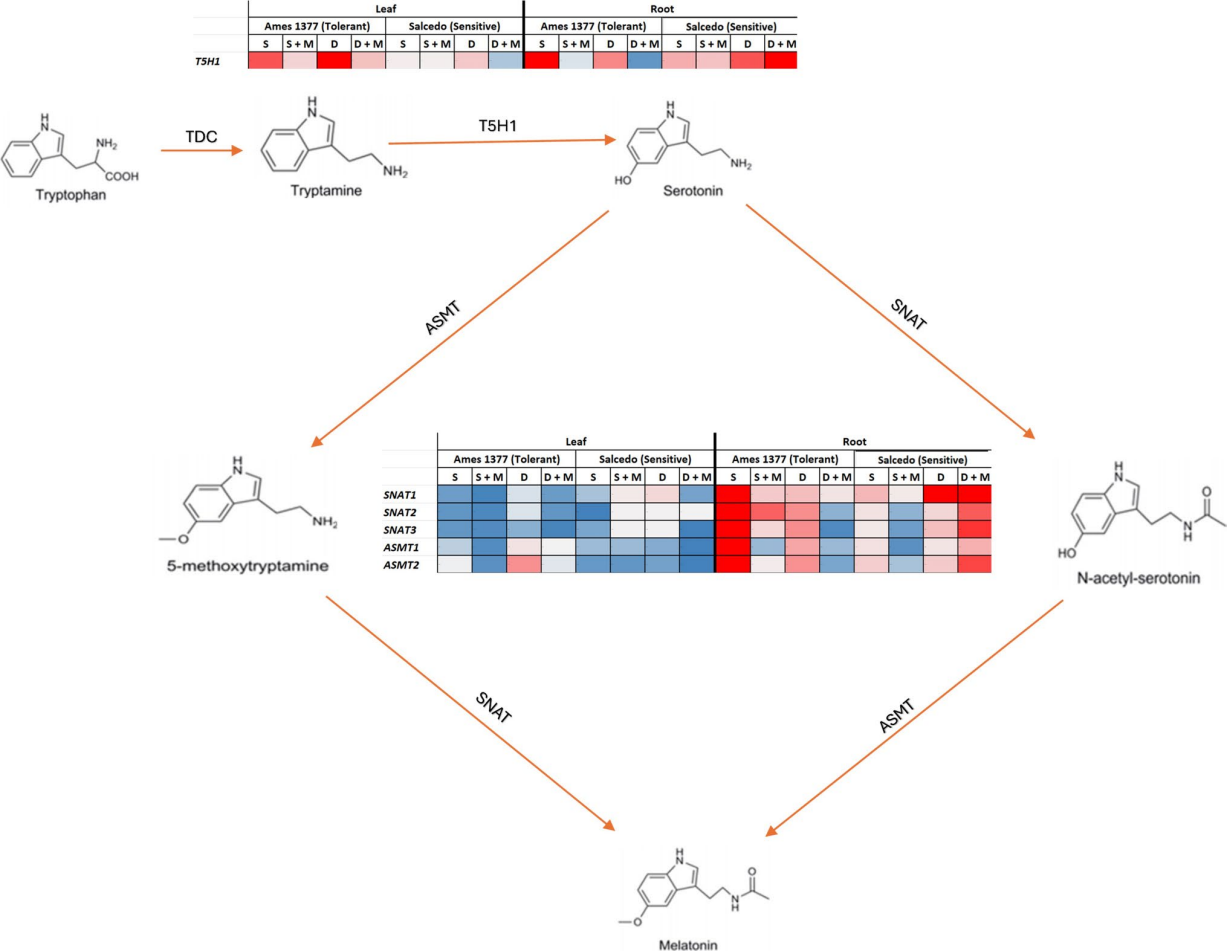
Representative transcription levels of the melatonin biosynthesis pathway genes in quinoa under salt and drought stress, with or without melatonin treatment (TDC: tryptophan decarboxylase, T5H: tryptamine 5-hydroxylase, SNAT: serotonin N-acetyltransferase, ASMT: N-acetylserotonin O-methyltransferase)

Here, in addition to in silico promoter analysis, the shoot length and physiological parameters, and expression profiles of *T5H1, SNAT1, SNAT2, SNAT3, ASMT1*, and *ASMT2* genes were investigated under drought and salt stress in two quinoa genotypes, Ames 1377 (stress-tolerant) and Salcedo (stress-sensitive). Under salt and drought stress conditions, the genes *T5H1, SNAT1, SNAT2, SNAT3, ASMT1*, and *ASMT2* exhibit differential expression, suggesting their active involvement in the rapid stress response to mitigate oxidative damage. In contrast, genes like *T5H2, TDC1, TDC2,* and *TDC3* maintain consistent expression, indicating a role in baseline functions under normal conditions. Similarly, in soybean, melatonin application under drought stress leads to differential regulation of certain biosynthetic pathway genes, while others remain unchanged (Wei et al. 2015). The tolerant genotype Ames 1377 exhibited no evidence of physiological changes following melatonin application, aside from a reduction in RWC under both stress conditions and an increase in electrolyte leakage under salinity stress, which suggests that defining stress tolerance in quinoa is complex. Quinoa displays a wide range of adaptive responses to environmental stresses. Tolerance is not determined by a single physiological parameter but rather by an interplay of multiple traits, such as osmotic adjustment, ion homeostasis, antioxidant capacity, and gene expression patterns. For example, a decrease in RWC or an increase in electrolyte leakage, typically viewed as negative indicators, may be counterbalanced by other protective mechanisms (e.g., enhanced antioxidant defenses or stress‐responsive gene activation) that help the plant maintain yield under adverse conditions (Hinojosa et al. 2018). This complexity means that relying on one or two markers may not accurately reflect overall tolerance. Figure 11 presents melatonin biosynthesis pathway, and the transcript levels of their corresponding genes in quinoa under salt and drought stress, with or without exogenous melatonin. The *T5H1* expression was upregulated in response to drought and salinity in both genotypes. However, its induction was significantly stronger in stress-tolerant Ames 1377 leaves compared to Salcedo. Notably, *T5H1* expression was markedly higher under drought stress than under salt treatment in both genotypes, suggesting that the serotonin biosynthesis pathway is specifically activated by drought conditions. A similar trend was observed in green foxtail (*Setaria viridis*) leaves, where its expression was significantly upregulated under drought and salt stress, with melatonin further enhancing this increase (Dangol et al. 2022). However, exogenous melatonin application mitigated this up-regulation, restoring gene expression to the control levels, especially in Salcedo. In contrast, while *T5H1* expression in Ames 1377 was significantly reduced following melatonin treatment, it did not return to control levels. This suggests that the genotype maintains a basal level of stress tolerance, preventing complete downregulation of the gene. *SNAT* genes were expressed at significantly high levels in the roots of the Ames 1377 compared to leaves under both stress conditions. This may suggest a root-centric mechanism for managing these abiotic stresses, likely through enhanced melatonin biosynthesis to mitigate salt/drought stress and improve plant tolerance. Roots, as the primary interface with environmental stressors like high salinity and water deprivation, play a critical role in the adaptive response to environmental stress conditions (Kalra et al. 2024). The enhanced expression of *SNAT1, SNAT2*, and *SNAT3* in the roots of Ames 1377 under stress conditions is correlated with the inherent tolerance of genotype, where stress conditions did not affect the shoot length, RWC, and electrolyte leakage of Ames 1377 as much as in sensitive Salcedo genotype. Upon melatonin application, the Salcedo genotype exhibited improved stress tolerance, leading to a reduction in physiological stress. However, the reduced expression of *SNAT* genes under stress in Ames 1377 leaves could indicate that melatonin is produced in the roots and then distributed to other tissues, including leaves. The transportation of melatonin to distal organs acting as an antioxidant in transported tissues is previously reported in response to cold stress in watermelon (*Citrullus lanatus* L.) (Li et al. 2017). Alternatively, if melatonin does reach the leaves, it might suppress the biosynthesis pathway through a negative feedback mechanism, wherein elevated melatonin levels inhibit further production to maintain homeostasis (Xie et al. 2023). In addition, the melatonin biosynthesis pathway may not be activated after melatonin application in quinoa leaves. Instead, ROS-scavenging pathways could be more prominently activated, correlating with the previous findings (Iqbal et al. 2018; Zhu et al. 2024). To further support our hypothesis, studies showed that exogenous melatonin enhances antioxidant enzyme activities, mitigating oxidative stress by effectively scavenging ROS in plants under abiotic stress (Gu et al. 2022). Conversely, the sensitive genotype Salcedo exhibited a less pronounced increase in expression of *SNAT* genes in the roots. This might be due to limited capacity of genotype to adapt to stress conditions which is supported by decreased shoot length and RWC under stress conditions. In general, exogenous melatonin application restored gene expression to the control levels and even reduced it further in sensitive Salcedo. Taken together, these results suggest that *SNAT* genes are predominantly expressed in the roots, where they likely contribute to stress adaptation. Their expression is suppressed by exogenous melatonin application in both roots and leaves, with a more pronounced downregulation in leaves, potentially to balance endogenous melatonin levels and fine-tune the plant's physiological response to stress. ASMT1 and ASMT2 are essential in melatonin biosynthesis and exhibit increased activity under abiotic stress conditions (Park et al. 2013). *ASMT1* and *ASMT2* expressions were upregulated under both stresses in root tissues of Ames 1377 and Salcedo similar to expression of *SNAT* genes. A previous study showed that *MzASMT1* overexpression increased tolerance against drought stress in *Arabidopsis*, and this regulation is further enhanced by melatonin application (Zuo et al. 2014). Furthermore, RWC values increased in both genotypes under both stresses after exogenous melatonin application compared to stress groups without melatonin. This supports that melatonin application increases the expression of *ASMT* genes and enhances plant tolerance against osmotic stress. In contrast, in leaf tissues, *ASMT1* and *ASMT2* expressions decreased under both stresses in Salcedo and under salt stress in Ames 1377. This downregulation might indicate that prolonged stress conditions overwhelm the quinoa’s ROS-scavenging capacity, leading to irreversible cellular damage as seen in electrolyte leakage results of Salcedo under salt stress.

Taken together, our gene expression results indicate that exogenous melatonin application contributes to stress resilience in quinoa, especially in the sensitive genotype, by modulating the expression of key genes involved in the melatonin biosynthesis. Collectively, expression patterns reveal *ASMT2* and *T5H1* as prime molecular targets for breeding stress-tolerant varieties, with *ASMT2* particularly promising due to its strong, consistent genotype × stress interactions across all tissues. These data indicate that while all genes respond to stress, their genetic regulation and tissue-specificity vary substantially, suggesting complementary roles in fine-tuning melatonin-mediated stress protection. The effects of melatonin can vary depending on plant species, cultivar, and environmental conditions, adding another layer of intricacy to its function. Additionally, interaction of melatonin with polyamines, JA, and proline metabolism according to GO enrichment analysis reveals its broader role in hormonal crosstalk and stress adaptation. These results suggest that melatonin in quinoa primarily functions as a regulator of stress responses—integrating antioxidant defense, hormonal signaling, and metabolic adjustments—rather than merely enhancing its own biosynthesis. This multi-layered regulatory mechanism underscores melatonin’s potential as a key target for engineering improved stress resilience in crops.

## Conclusion

Melatonin biosynthesis pathway is known to have four enzymes, including tryptophan decarboxylase (TDC), tryptamine 5-hydroxylase (T5H), serotonin N-acetyltransferase (SNAT), and N-acetylserotonin O-methyltransferase (ASMT). The objective of this study was to identify and characterize quinoa genes involved in melatonin biosynthesis pathway, and analyze growth, physiological responses, and expression levels of genes in Salcedo and Ames 1377 genotypes under drought and salt stress.

In the present study, we identified 3 *TDCs*, 2 *TSHs*, 3 *SNATs*, and 2 *ASMT genes* in quinoa genome for the first time. The protein lengths of melatonin biosynthesis enzymes varied from 105 (CqSNAT3) to 635 (CqTDC2) amino acids, while their pI values ranged from 4.91 (SNAT1) to 9.69 (SNAT2). PlantCARE tool showed that the *cis-*elements in *TDC, T5H,* and *SNAT* promoters were mostly associated with stress response, while those in *ASMT* promoters were related to light response. Additionally, interaction of melatonin with polyamines, jasmonic acid (JA), and proline metabolism according to GO enrichment analysis reveals its broader role in hormonal crosstalk and stress adaptation. Exogenous melatonin application mitigated the negative effects of salt and drought stress on shoot length, RWC, and electrolyte leakage in the sensitive Salcedo genotype. In contrast, its protective role against stress was not significant in the stress-tolerant Ames 1377 genotype. Under drought and salt stress, *T5H1, SNAT1, SNAT2, SNAT3, ASMT1,* and *ASMT2* showed differential expression levels between Ames 1377 and Salcedo. In Ames 1377, these genes were highly upregulated in roots, might be suggesting a root-centric melatonin biosynthesis strategy for ROS scavenging and osmotic balance, while Salcedo exhibited lower root expression, indicating a weaker stress response. After exogenous melatonin application, gene expression patterns changed. In Ames 1377, expression of *SNAT* and *ASMT* further decreased, suggesting that melatonin biosynthesis was not activated, and instead, other pathways resulting in ROS scavenging and proline accumulation might predominate for increased tolerance. In contrast, Salcedo benefited more from melatonin application, as it helped mitigate stress-induced damage and normalize gene expression. This study comprehensively characterized melatonin biosynthetic genes, and their expression patterns in response to salt and drought in quinoa, providing preliminary information for further studies on improvement of stress tolerance in crops.

## Supplementary Information

Below is the link to the electronic supplementary material.

Supplementary file1 (DOCX 25 KB)

Supplementary file2 (XLSX 23 KB)

## Data Availability

The TDC, T5H, SNAT, and ASMT data from different plant species presented in this study can be found in National Center for Biotechnology Information (NCBI), Ensembl Plants, The Arabidopsis Information Resource (TAIR), and Phytozome version 13. All data analyzed in this study are already included in the manuscript.
