# Supplementary material for: Deciphering melatonin biosynthesis pathway in Chenopodium quinoa: genome-wide analysis and expression levels of the genes under salt and drought
Source: Planta. 2025 Jun 12;262(1):23. doi: 10.1007/s00425-025-04741-x (PMC12162789; doi:10.1007/s00425-025-04741-x)
Supplement: Supplementary file 1 — Supplementary file1 (DOCX 25 KB) [file 425_2025_4741_MOESM1_ESM.docx]

**Table S1.** The list of *Arabidopsis* orthologs of *SNAT1, SNAT2, SNAT3, ASMT1, ASMT2,* and *T5H1* genes from *C. quinoa*, which were used to search the PPI networks through String version 12.0.

|  | ***Arabidopsis* Ortholog** | | |
| --- | --- | --- | --- |
| **Quinoa Gene ID** | **AGI** | **Gene Name** | **Gene Abbreviation** |
| XP_021726561 | AT4G28680 | *L-TYROSINE DECARBOXYLASE 1* | *TYRDC1* |
| XP_021730824 | AT4G28680 | *L-TYROSINE DECARBOXYLASE 1* | *TYRDC1* |
| XP_021746001 | AT4G28680 | *L-TYROSINE DECARBOXYLASE 1* | *TYRDC1* |
| XP_021719925 | AT3G48270 | *CYTOCHROME P450, FAMILY 71, SUBFAMILY A, POLYPEPTIDE 26* | *CYP71A26* |
| XP_021749809 | AT3G48270 | *CYTOCHROME P450, FAMILY 71, SUBFAMILY A, POLYPEPTIDE 26* | *CYP71A26* |
| XP_021767414 | AT1G32070 | *SEROTONIN N-ACETYLTRANSFERASE 1* | *SNAT1* |
| XP_021718295 | AT1G26220 | *SEROTONIN N-ACETYLTRANSFERASE 2* | *SNAT2* |
| XP_021723321 | AT1G32070 | *SEROTONIN N-ACETYLTRANSFERASE 1* | *SNAT1* |
| XP_021774904 | AT4G35160 | *N-ACETYLSEROTONIN O-METHYLTRANSFERASE* | *ASMT1* |
| XP_021753002 | AT4G35160 | *N-ACETYLSEROTONIN O-METHYLTRANSFERASE* | *ASMT1* |

**Table S2.** The gene clusters which were used as a set to determine the gene ontology (GO) enrichment for biological process and molecular function.

**Table S3.** Quinoa genes and primers used in qRT-PCR analysis.

| **Gene name** | **Forward primer** | **Reverse primer** |
| --- | --- | --- |
| *SNAT1* | GGAAGAGCCTTTGCCTGAA | TCACATAATGCCTGTAGGTCA |
| *SNAT2* | TCTTCCATCACCCTCCCTCC | TGAGACCATCAGTGTTGCGG |
| *SNAT3* | GCATTCTGTTCGTAAATCACCTG | GAGCCATTCCAATGAGCGTC |
| *ASMT1* | TTCCAAAAGCCGATGTTGC | CTCTACAATGATTACCTTCCC |
| *ASMT2* | TGAAGAGGCACAAGCAAGTG | AGGGAAGAGGCTAGTTCGGT |
| *T5H1* | TGGAACAGAAGGCGGTATCA | TCTTTGGCAACCCTGTCAACT |
| *ACTIN1* | CGTGACCTGACAGACCACTT | CCCATCGGGGAGTTCATAGC |

**Table S6.** Two-way ANOVA results for shoot length, relative water content and electrolyte leakage.

| **Source** | **SS** | **DF** | **MS** | **F-value** | ***p*-value** |
| --- | --- | --- | --- | --- | --- |
| **Shoot length** | | | | | |
| Genotype | 682.3 | 1 | 682.3 | 82.37 | <0.001 |
| Treatment | 628.9 | 4 | 157.2 | 18.92 | <0.001 |
| Genotype×Treatment | 134.6 | 4 | 33.7 | 4.05 | 0.007 |
| Residual (Error) | 448.1 | 54 | 8.3 | – | – |
| **Relative water content** | | | | | |
| Genotype | 385.7 | 1 | 385.7 | 12.6 | 0.001 |
| Treatment | 2216.4 | 4 | 554.1 | 18.1 | <0.001 |
| Genotype×Treatment | 612.8 | 4 | 153.2 | 5 | 0.002 |
| Residual (Error) | 1040.9 | 34 | 30.6 | – | – |
| **Electrolyte leakage** | | | | | |
| Genotype | 6854.4 | 1 | 6854.4 | 54.6 | <0.001 |
| Treatment | 6059.1 | 4 | 1514.8 | 12.1 | <0.001 |
| Genotype×Treatment | 793.4 | 4 | 198.3 | 1.6 | 0.2 |
| Residual (Error) | 3639.8 | 29 | 125.5 | – | – |

SS: sum of squares; MS: mean squares; DF: degrees of freedom
